# The *Drosophila junctophilin* gene is functionally equivalent to its four mammalian counterparts and is a modifier of a Huntingtin poly-Q expansion and the Notch pathway

**DOI:** 10.1242/dmm.029082

**Published:** 2018-01-01

**Authors:** Eduardo Calpena, Víctor López del Amo, Mouli Chakraborty, Beatriz Llamusí, Rubén Artero, Carmen Espinós, Máximo I. Galindo

**Affiliations:** 1 Program in Molecular Mechanisms of Disease, Centro de Investigación Príncipe Felipe (CIPF), c/ Eduardo Primo Yúfera no. 3, 46012 Valencia, Spain; 2 Translational Genomics Group, Incliva Health Research Institute, Avda. Menendez Pelayo 4 acc 46010, Valencia, Spain; 3 Department of Genetics and Estructura de Recerca Interdisciplinar en Biotecnologia i Biomedicina (ERI BIOTECMED), Universitat de València, c/ Dr Moliner 50, 46100 Burjasot, Spain; 4 UPV-CIPF Joint Unit Disease Mechanisms and Nanomedicine, 46012 Valencia, Spain; 5 Instituto Interuniversitario de Investigación de Reconocimiento Molecular y Desarrollo Tecnológico (IDM), Universitat Politècnica de València, Universitat de València, 46022 Valencia, Spain

**Keywords:** Junctophilin, *Drosophila*, Cardiomyopathy, Huntington's disease, Notch

## Abstract

Members of the Junctophilin (JPH) protein family have emerged as key actors in all excitable cells, with crucial implications for human pathophysiology. In mammals, this family consists of four members (JPH1-JPH4) that are differentially expressed throughout excitable cells. The analysis of knockout mice lacking JPH subtypes has demonstrated their essential contribution to physiological functions in skeletal and cardiac muscles and in neurons. Moreover, mutations in the human *JPH2* gene are associated with hypertrophic and dilated cardiomyopathies; mutations in *JPH3* are responsible for the neurodegenerative Huntington's disease-like-2 (HDL2), whereas *JPH1* acts as a genetic modifier in Charcot–Marie–Tooth 2K peripheral neuropathy. *Drosophila melanogaster* has a single *junctophilin* (*jp*) gene, as is the case in all invertebrates, which might retain equivalent functions of the four homologous JPH genes present in mammalian genomes. Therefore, owing to the lack of putatively redundant genes, a *jp* *Drosophila* model could provide an excellent platform to model the Junctophilin-related diseases, to discover the ancestral functions of the JPH proteins and to reveal new pathways. By up- and downregulation of Jp in a tissue-specific manner in *Drosophila*, we show that altering its levels of expression produces a phenotypic spectrum characterized by muscular deficits, dilated cardiomyopathy and neuronal alterations. Importantly, our study has demonstrated that Jp modifies the neuronal degeneration in a *Drosophila* model of Huntington's disease, and it has allowed us to uncover an unsuspected functional relationship with the Notch pathway. Therefore, this *Drosophila* model has revealed new aspects of Junctophilin function that can be relevant for the disease mechanisms of their human counterparts.

## Abstract (precis)

**Summary:** This work reveals that the *Drosophila* Junctophilin protein has similar functions to its mammalian homologues and uncovers new interactions of potential biomedical interest with Huntingtin and Notch signalling.

## INTRODUCTION

Junctophilin (JPH) family proteins contribute to the formation and maintenance of junctional membrane complexes (JMCs) by serving as a physical bridge between the plasma membrane (PM) and the endoplasmic reticulum (ER) membrane in excitable cells, allowing the functional crosstalk between ion channels (Takeshima et al., 2015, 2000). Silencing or genetic ablation of the JPH genes produces defects in Ca^2+^ homeostasis (Hirata et al., 2006; Ito et al., 2001; Li et al., 2010; Moriguchi et al., 2006; Takeshima et al., 2015, 2000). In mammals, this family comprises four members (*JPH1-JPH4*) that are differentially expressed: *JPH1* is predominantly expressed in skeletal muscle, *JPH2* in skeletal muscle and heart, and *JPH3* and *JPH4* genes are coexpressed in neural tissues (Nishi et al., 2000, 2003; Takeshima et al., 2000).

*Jph1* knockout (KO) mice exhibit suckling failure and die shortly after birth with morphological and physiological abnormalities in skeletal muscle, including fewer JMCs, failure of normal triad development, abnormal ER features and reduced contractile force (Ito et al., 2001; Komazaki et al., 2002). Jph2 plays a key role in cardiomyocyte development and stability of myocyte ultrastructure (Beavers et al., 2014; Chen et al., 2013; Reynolds et al., 2013; Takeshima et al., 2000). *Jph2* null mice die during the early embryonic development, as a result of cardiac failure (Takeshima et al., 2000). Cardiac myocytes from these mutant mice showed deficiency of the JMCs and abnormal structures in ER and mitochondria. Mice with decreased levels of cardiac *Jph2* showed defective postnatal T-tubule maturation, whereas mice overexpressing *Jph2* had accelerated T-tubule maturation (Reynolds et al., 2013). Inducible cardiac-specific *Jph2* knockdown in mice leads to ventricular dilatation, postnatal heart failure and increased mortality (Reynolds et al., 2013).

*Jph3* null mice exhibit adult onset, progressive motor dysfunction, whereas *Jph3* hemizygous mice have a similar but milder phenotype (Seixas et al., 2012). Knockout mice lacking *Jph4* show no obvious abnormalities, suggesting functional redundancy between Jph3 and Jph4 (Moriguchi et al., 2006). Double KO mice lacking both *Jph3* and *Jph4* genes have severe growth retardation and die within 3-4 weeks after birth, probably as a result of impairment of the neuronal circuit controlling the salivary gland (Moriguchi et al., 2006). In addition, they exhibit impaired motor coordination, learning and memory (Ikeda et al., 2007; Kakizawa et al., 2008; Moriguchi et al., 2006).

*JPH* genes have been found to play important roles in pathology. In an *mdx* mouse model of Duchenne muscular dystrophy, aberrant Jph1 proteolysis was detected, providing an association with the development of primary muscle disease (Murphy et al., 2013). JPH2 dysregulation has been associated with variety of heart diseases (Landstrom et al., 2014; Takeshima et al., 2015). Decreased levels of *Jph2* expression have been reported in animal models of aortic stenosis (Xu et al., 2007) and hypertrophic and dilated cardiomyopathy (Minamisawa et al., 2004). In addition, in human failing heart samples or in patients with hypertrophic cardiomyopathy, the *JPH2* levels are markedly reduced (Landstrom et al., 2011; Zhang et al., 2013). Importantly, dominant mutations in the human *JPH2* gene are associated with hypertrophic and dilated cardiomyopathy (Beavers et al., 2013; Landstrom et al., 2007; Sabater-Molina et al., 2016) and constitute a relatively rare cause of congenital cardiomyopathies.

Huntington's disease-like 2 (HDL2) is a rare, autosomal dominant neurodegenerative disorder that is clinically almost indistinguishable from Huntington's disease (HD). HDL2 is caused by a CTG/CAG expansion located within the alternatively spliced exon 2A of the *JPH3* gene (Holmes et al., 2001). The pathogenicity of this mutation involves both a toxic gain of function attributable to the expansion and a reduction in the levels of JPH3 protein expression (Seixas et al., 2012). Recently, *JPH1* has been described as a genetic modifier for the Charcot–Marie–Tooth 2K (CMT2K) peripheral neuropathy (Pla-Martin et al., 2015).

*Drosophila melanogaster* has a single *junctophilin* (*jp*) gene, as is the case in all invertebrate genomes (Garbino et al., 2009; Takeshima et al., 2015), which can be an advantage for study of the molecular function of this protein family. In addition to the power of genetic analysis in *Drosophila*, the presence of a single *jp* gene will prevent masking of the mutant phenotype by other family members, as has been described for murine *Jph3* and *Jph4*. The restricted tissue specificity of the different JPH genes in mammals is not completely clear cut: JPH1 is required in peripheral nerves in addition to its more studied role in muscle (Pla-Martin et al., 2015); and the neural JPH3 and JPH4 proteins are also required in pancreatic β cells and T cells, respectively (Li et al., 2016; Woo et al., 2016). Therefore, an animal model with a single *junctophilin* gene can help to uncover more ancestral functions. We decided to investigate the phenotypic spectrum of altering *jp* in *Drosophila* in order to find out whether it also reproduces histological alterations compatible with those found in KO mice lacking *Jph* subtypes and in patients with mutations in JPH genes; and to reveal new aspects of Junctophilin function that can be relevant for the disease mechanisms of their human counterparts.

## RESULTS

### Generation of the overexpression and RNA interference models

We decided to generate models for overexpression (OE) and knockdown (KD) of *jp* to investigate their effects on the target tissues. Both conditions are based on the Gal4/UAS system for directed expression (Brand and Perrimon, 1993), where a tissue-specific *Gal4* construct drives expression of another transgenic construct under the *UAS* promoter.

**Fig. 1.**
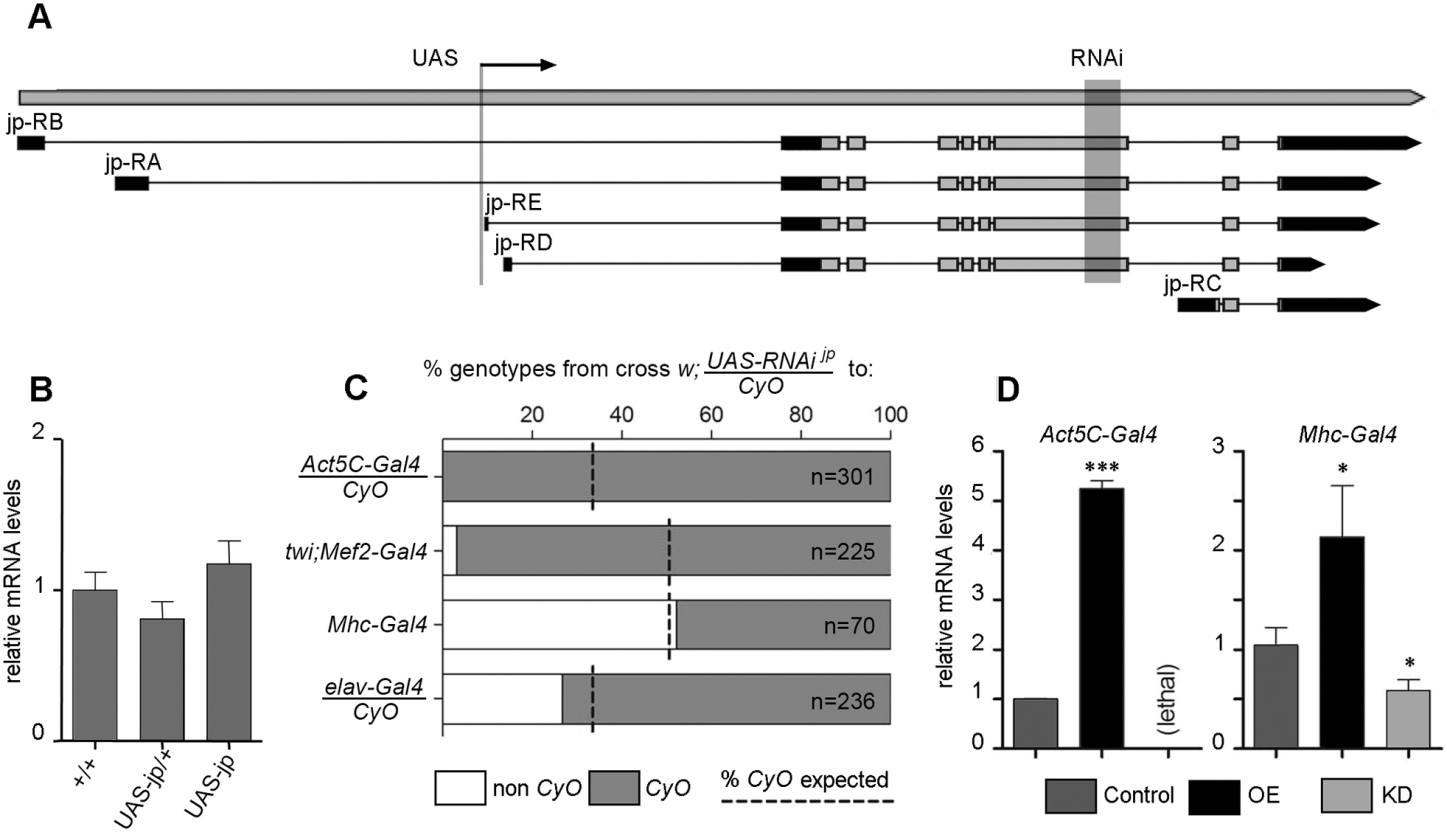
**Generation and validation of stocks for overexpression and RNAi.** (A) Schematic representation of the *jp* locus, with gene span, structure of the five isoforms identified (coding region light grey), insertion site of the *P{XP}jp^d04563^* construct (UAS, arrow) and target region of the RNAi line *P{KK107921}VIE-260B* (RNAi, shaded). (B) mRNA levels of the *jp* gene in *jp^+^* wild-type *Oregon-R* flies, and in heterozygotes and homozygotes for the *UAS-jp* insertion do not show any significant differences. (C) Survival of the progeny expressing the *jp* RNAi driven by each one of the four *Gal4* drivers tested. The dashed line indicates the expected proportion of control flies expected (mendelian proportions 1:1 or 1:2) and the white portion of the bar the proportion observed. (D) Relative mRNA levels of *jp* in the OE and KD genotypes generated by crossing to two different *Gal4* lines compared with the control flies bearing only the *Gal4* construct. In bar diagrams, data are represented as the mean±s.e.m. One-way ANOVA, *P<0.05, ***P<0.001.

The single *Drosophila junctophilin* gene (*jp*, initially annotated *CG4405*) is homologous to the four human JPH genes (*JPH1-JPH4*), which originated from a single ancestral gene by successive duplications (Garbino et al., 2009). High-throughput studies in *Drosophila* have shown that *jp* is expressed in both muscular and neural tissues (Chintapalli et al., 2007; Kumar et al., 2011); data available from http://flybase.org. The *jp* gene produces five different transcripts that differ in their transcription start sites, four of which produce the same 1054-amino acid-long open reading frame coding for the canonical Jp protein (Fig. 1A). The fifth transcript, coding for a 129-amino acid-long open reading frame, is probably spurious and definitely nonfunctional, because it lacks most functional domains. To generate a *UAS-jp*, we obtained a stock with an insertion of the *P{XP}* transgene within the *jp* locus, *P{XP}jp^d04563^*. This *P{XP}* transgenic construct is inserted upstream of the first coding exon (Fig. 1A) and has two *UAS* promoters, one at each end and in different orientations, so that the right promoter points towards the *jp* gene and the left promoter towards the upstream region (Thibault et al., 2004). This second promoter is flanked by *FRT* recombination sites, so it was removed by crossing to an *FLP* transgene, leaving only the *jp*-specific UAS promoter. Details of this process are given in Fig. S1. This insertion will be referred to as *UAS-jp*. An advantage of this *UAS* line compared with the ones generated by random insertion is that, as it is already inserted in the target locus, it does not produce insertional mutations in other genes. As the insertion is viable in homozygosis, most probably it does not affect expression of the *jp* gene substantially, but we cannot discard the possibility that it partly hinders expression and therefore it could be a mild hypomorphic allele. In order to test this possibility, we quantified the *jp* mRNA levels in individuals heterozygous and homozygous for the insertion. This analysis revealed no statistically significant differences with *Oregon-R* wild-type flies (Fig. 1B); thus, the insertion does not affect *jp* transcript expression levels and therefore it is not a hypomorph.

For RNA interference (RNAi), we obtained a stock from the Vienna *Drosophila* Resource Centre collection (Dietzl et al., 2007) that contains the insertion *P{KK107921}VIE-260B*, whose target sequence is contained in an exon that is common to the four major isoforms of the *jp* gene (Fig. 1A). This line is described as having one on-target and no off-target sites, and has the maximum s_19_ specificity score (the number of 19-mer on-target matches divided by the total number of matches) of one (Dietzl et al., 2007) and only three CAN repeats, which is below the desired threshold of six repeats (Ma et al., 2006). In addition, the KK collection was generated by insertion of the *UAS-RNAi* constructs into fixed attP sites in order to achieve reproducible expression levels and also avoid position effects and random insertional mutagenesis. This insertion will be referred to as *UAS-jp^RNAi^*.

To validate both constructs, we crossed them to different *Gal4* lines that drive expression ubiquitously (*Ac5C-Gal4/CyO*) or specifically in muscular [*twi;Mef2-Gal4 (II)*, early mesoderm and derivatives; *Mhc-Gal4 (II)*, differentiated muscle] or nervous tissues (*elav-Gal4/CyO*, post-mitotic neurons). As the *twi;Mef2-Gal4* and *Mhc-Gal4* insertions are homozygous viable, if the cross with one of the *UAS* lines was lethal we would observe no progeny, making it difficult to assess whether this was true lethality or attributable to a crossing failure. For this reason, we used both *UAS* lines over a *CyO* chromosome; even if the cross were lethal, we should observe several *CyO* flies.

All four gave a viable progeny when crossed to *UAS-jp*, and so did the combination of *UAS-jp^RNAi^* with the late drivers *Mhc-Gal4* and *elav-Gal4*; but the combination of *UAS-jp^RNAi^* with early expression drivers *Ac5C-Gal4* and *twi;Mef2-Gal4* was lethal (Fig. 1C). This suggests that the *jp* gene is strictly required for development and/or cell survival. We tried other ubiquitous lines and lower culture temperatures, but all attempts resulted in lethality during embryonic or early larval development.

Next, we tested that the *UAS-jp* and *UAS-jp^RNAi^* lines had the expected effect on the levels of *jp* transcript, by means of quatitative PCR. With the strongest line, *Act5C-Gal4*, we obtained a fivefold increase in *jp* transcript levels with the *UAS-jp* construct (Fig. 1D), but because the cross to *UAS-jp^RNAi^* was lethal, we could not perform the quantification in this condition. To validate the *UAS-jp^RNAi^* construct, we dissected thoraxes of *Mhc-Gal4/UAS-jp^RNAi^*, where muscle tissue is predominant, and we observed a significant decrease in *jp* levels (Fig. 1D). This decrease is an underestimation of the real one, because the thorax contains other tissues where Mhc-Gal4 is not expressed, such as the thoracic ganglion of the central nervous system. Further proof of the specificity of this RNAi construct is the fact that it can compensate for overexpression of *jp* in different tissues (see the results of expression in retina and wing below). We used these *Drosophila* transgenic lines to analyse the effect of altering the levels of Jp in tissue-specific OE and KD conditions, by comparison with control flies bearing the same *Gal4* driver but no UAS construct.

### Muscular deficits in the *Drosophila* Jp models

**Fig. 2.**
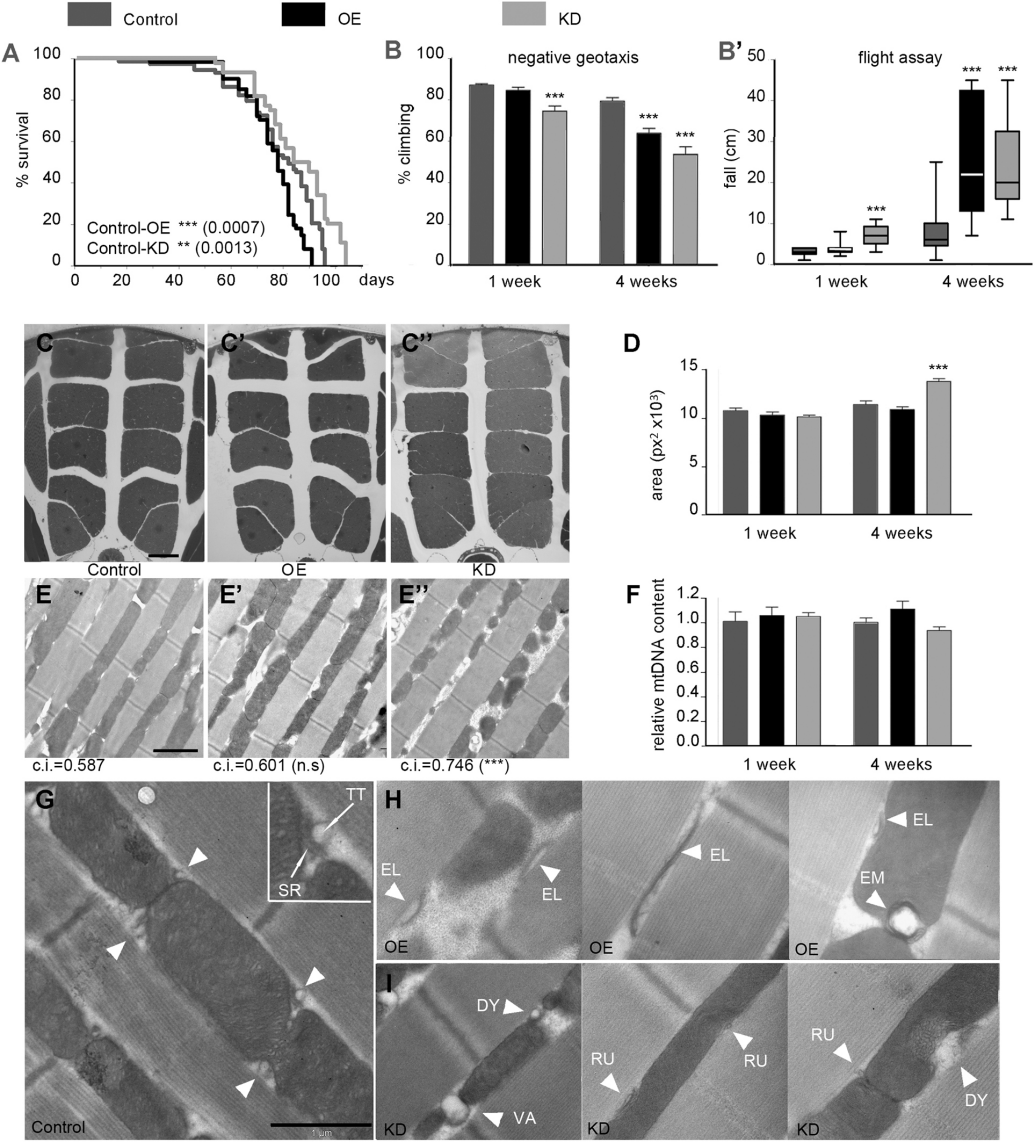
**Muscular deficits in OE and KD flies.** (A) Survival curves of the control (*Mhc-Gal4*), OE and KD flies; log-rank (Mantel–Cox) test shows significant differences from the control genotype. (B,B′) Neuromuscular competence of the three genotypes at 1 and 4 weeks of age estimated in the negative geotaxis assay (B, *n*=3≥15 flies each) and in the flight assay (B′, *n*=30). (C-C″) Semi-thin sections of the thorax of control (C), OE (C′) and KD (C″) flies at 4 weeks of age. (D) Area occupied by the IFMs in semi-thin sections at 1 and 4 weeks of age (*n*≥6, three sections analysed per individual). (E-E″) TEM of ultra-thin longitudinal sections of the IFMs of control (E), OE (E′) and KD (E″) flies at 4 weeks of age; under each panel the mitochondrial circularity index (c.i.) is indicated (*n*=3, 60 mitochondria per individual). (F) Relative mitochondrial genomic DNA; in each genotype and age the proportion of mitochondrial to nuclear genomic DNAs was calculated, and the results are displayed as mitochondrial genomic DNA abundance relative to the control genotype at each age. (G) Muscle fibre ultrastructure in a 1-week-old control fly. Diads are indicated by white arrowheads, representing the normal morphology of these structures. The inset shows a wild-type diad with indication of the sarcoplasmic reticulum (SR) and T-tubule (TT) components. (H) Examples of aberrant morphology, such as elongated SR cisternae (EL) and diads embedded in the mitochondria (EM), in 1-week-old OE fly muscles. (I) Abnormal rudimentary (RU), dysmorphic (DY) or vacuolated (VA) diads (arrowheads) in 1-week-old KD fly muscles, usually next to disorganised mitochondrial cristae. Scale bars: 100 μm in C-C″; 2 μm in E-E″; 1 μm in G-I. In bar diagrams, data are represented as mean±s.e.m., One-way ANOVA,**P<0.01, ***P<0.001.

In mammals, JPH1 is the major JPH family member expressed in skeletal muscle (Ito et al., 2001; Komazaki et al., 2002). In *Drosophila*, as we mentioned above, silencing of *jp* with the early mesoderm-specific driver *twi;Mef2-Gal4* resulted in lethality, so we had to use the muscle-specific *Mhc-Gal4* driver that produced viable adults (Fig. 1B). Our control genotype was *Mhc-Gal4/+*, the OE genotype *Mhc-Gal4/UAS-jp*, and the KD genotype *Mhc-Gal-4/UAS-jp^RNAi^*. Regarding longevity, OE flies had only a slight reduction in viability, whereas KD flies had an extension of the maximal lifespan of ∼10 days (Fig. 2A). The possible role of insulin signalling downregulation in this extended lifespan is explored in the Discussion section. In order to measure the muscular competence, we performed negative geotaxis and flight assays (Fig. 2B,B′). In both tests, the KD flies already showed a markedly decreased performance at 1 week of age; and both OE and KD genotypes had a more severe age-dependent reduction in the ability to climb the vial and to attain stable flight.

To determine whether this motor deficit is attributable to muscular degeneration, we analysed the indirect flight muscles (IFM) in semi-thin sections of the thorax. No significant differences were found at 1 week of age, but at 4 weeks horizontal gaps within each set of muscle packs are evidently reduced in KD flies compared with control and OE flies (Fig. 2C-C″), and this is attributable to an increase in the area of the IFM sections (Fig. 2D). This result suggests an age-dependent muscle hypertrophy in KD flies, which might be related to the defects observed in the climbing assay. To study the ultrastructure of IFM by transmission electron microscopy, we performed longitudinal sections along the muscle fibres. The structure of the myofibrils was not affected in any genotype, but KD flies displayed an aberrant mitochondrial morphology, which was most evident at 4 weeks (Fig. 2E-E″). These mitochondria were smaller and more rounded than in the wild-type control. Mitochondria from control and OE flies are similar, with a circularity index ∼0.6, whereas KD mitochondria are more circular, with an average index of 0.75, and these differences are statistically significant. Given that KD flies have abnormally shaped mitochondria, we estimated mitochondrial biomass by qPCR, to determine the mitochondrial genomic DNA content, using the nuclear genomic DNA for normalization, and we did not find any significant differences between OE or KD and the control.

Given that loss of Jph1 in skeletal muscle results in aberrant triads, we examined the morphology of the equivalent structure in the insect muscle, the diads, formed by a single electron-dense SR cisterna and the adjoining electron-lucent T-tubule (Razzaq et al., 2001). We performed this analysis at 1 week of age, before major morphological changes in mitochondria. Diads are located half-way between the Z and M bands of the myofibril (Fig. 2G). OE muscles have aberrant diads with elongated SR cisternae, and in mitochondria-poor regions unusually elongated SR cisternae are surrounded by electron-lucent material, which might be an expanded T-tubule structure (Fig. 2H). Occasionally, diads also show an abnormal positioning away from the myofibrils and embedded in the mitochondria. Diads in KD muscles have a different phenotype. They are rudimentary or have an aberrant morphology, including a vacuolated SR similar to the one in *Jph1* KO mice (Fig. 2I); and the adjoining mitochondrial regions also have abnormal cristae morphology.

Human *JPH1* is a modifier of the *GDAP1* mutations causing Charcot–Marie–Tooth peripheral neuropathy (Pla-Martin et al., 2015). Previous work from our group found metabolic alterations in *Drosophila Gdap1* models (López Del Amo et al., 2017). To determine whether altered levels of Jp result in metabolic changes in the muscle, we carried out a metabolomic study by nuclear magnetic resonance (NMR). In order to detect early and direct metabolic alterations that could contribute to reduced muscular competence, we performed this study in 1-week-old flies. We compared the control genotype with the OE and KD genotypes, and in both cases, we could find a discriminating model by orthogonal projection on latent structure-discriminant analysis (OPLS-DA) (Fig. S2), which means that the compared genotypes are metabolically different. The most marked change in both experimental genotypes is a significant increase in glycogen, the main carbohydrate storage species in the muscle. At the same time, there is a reduction in trehalose, which is the main source of energy for the IFM. Next, we paid attention to metabolites known to reflect the homeostasis of the muscle. The abundance of β-alanine is a marker of muscle degeneration (Sarou-Kanian et al., 2015), and levels of this metabolite had no significant changes. The three branched-chain amino acids (isoleucine, leucine and valine) promote protein synthesis in the muscle (Kimball and Jefferson, 2006), and their abundance was reduced only in KD flies. These results reinforce the notion that imbalanced *jp* levels affect muscle function but do not result in major muscular degeneration.

### Cardiac dysfunction produced by altered Jp expression

**Fig. 3.**
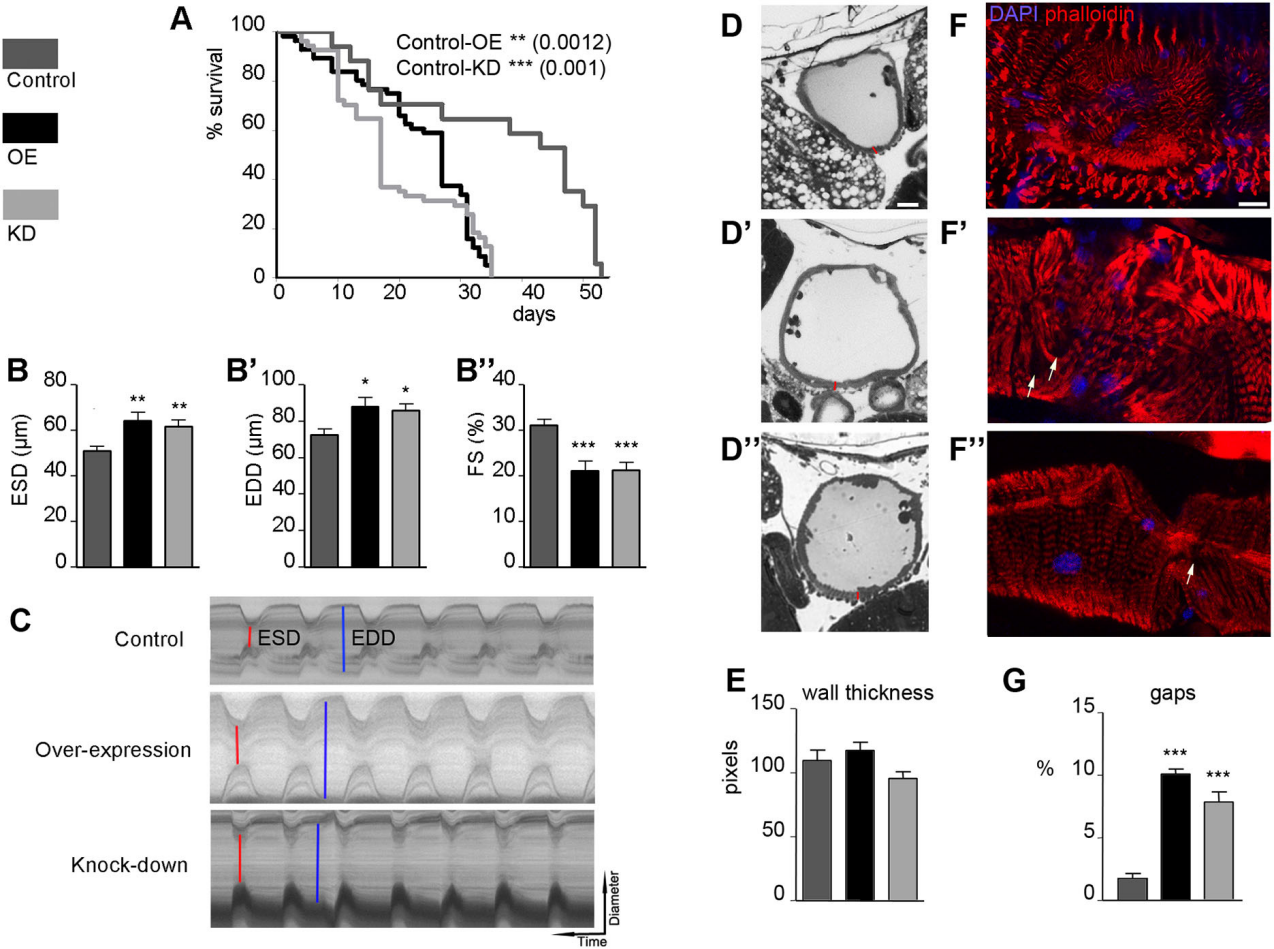
**Cardiac dysfunction in OE and KD flies.** (A) Survival curves of the control (*GMH5-Gal4*), OE and KD flies; log-rank (Mantel–Cox) test shows significant differences from the control genotype. (B-B″) Cardiac function parameters altered in the experimental genotypes at 1 week of age (*n*≥15 per genotype): end-systolic diameter (B), end-diastolic diameter (B′) and fractional shortening (B″). (C) Representative M-mode traces (vertical movement of the heart walls in 14 s) of semi-intact *Drosophila* hearts from the different genotypes; ESD are indicated in red and EDD in blue. (D-D″) Semi-thin sections of adult hearts of 1-week-old flies of the control (D), OE (D′) and KD (D″) genotypes. Red bars represent heart wall thickness measurements like the ones that have been used for the quantification in E. (E) Quantification of heart wall thickness in the three genotypes as mean pixels (*n*≥10, three measurements per sample). (F-F″) Phalloidin staining of dissected hearts with DAPI staining of nuclei reveals the normal myofibril structure in control 1-week-old flies (F) and abnormal morphologies in same age OE (F′) and KD (D″) hearts; white arrows point to gaps, areas devoid of myofibrils. (G) Quantification of the proportion of surface with gaps in the myofibrillar staining of five hearts of different genotypes (*n*=5). Scale bars: 10 μm in D-D″ and F-F″. In bar diagrams, data are represented as mean±s.e.m. One-way ANOVA, *P<0.05, **P<0.01, ***P<0.001.

**Fig. 4.**
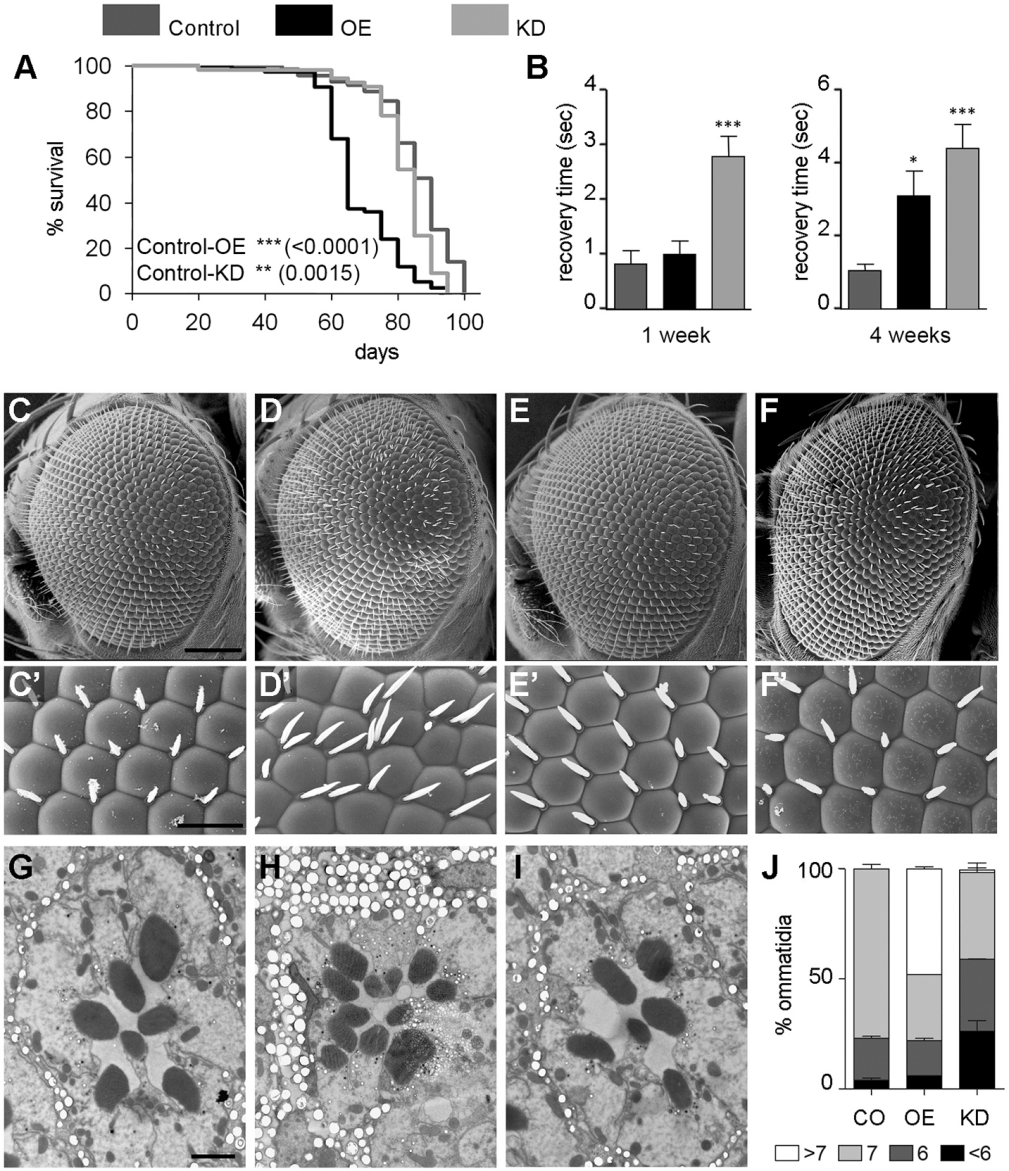
**Neuronal alterations in the OE and KD genotypes.** (A) Survival curves of the control (*elav-Gal4*), OE and KD flies; log-rank (Mantel–Cox) test shows significant differences with the control genotype. (B) Bang-sensitivity analyses of the three genotypes at 1 and 4 weeks of age, represented as recovery time after mechanical stress-induced paralysis. (*n*=4, ≥10 flies per experiment). (C,C′) SEM image of a wild-type control eye (*GMR-Gal4*, C) and higher magnification showing the stereotypical hexagonal arrangement of the ommatidia and the inter-ommatidial bristles (C′). (D,D′) In an OE fly, the eye has a rough aspect (D), and the ommatidia have lost the regular pattern and have supernumerary bristles (D′). (E,E′) KD fly eyes have a wild-type aspect (E) and structural arrangement (E′). (F,F′) The defects in *UAS-jp* eyes are corrected by coexpression of *UAS-jp^RNAi^*. (G) SEM of an ultra-thin section of a control fly ommatidium, showing the wild-type trapezoidal arrangement with seven rhabdomeres. (H) Structure of an ommatidial section of an OE fly with extra rhabdomeres, as a result of the recruitment of extra photoreceptor neurons. (I) Loss of rhabdomeres, hence photoreceptor neurons, in a KD ommatidial section. (J) Distribution of the number photoreceptor neurons in ommatidia of the three genotypes at 1 week of age (*n*=4, ≥60 ommatidia per section). Scale bars: 100 μm in C-F; 20 μm in C′-F′; 2 μm in G-I. In bar diagrams, data are represented as mean±s.e.m. One-way ANOVA, *P<0.05.

Constitutive or heart-specific loss of *Jph2* produces cardiac defects in mice (Takeshima et al., 2000). In order to investigate the relevance of Jp in the *Drosophila* heart, we used the cardiac-specific driver *GMH5-Gal4*. All the results we show below have been obtained with a culture temperature of 29°C, at which the cardiac phenotypes were most evident. In this case, our control genotype was *GMH5-Gal4/+*, the OE genotype *GMH5-Gal4/UAS-jp*, and the KD genotype *GMH5-Gal4/UAS-jp^RNAi^*. Both interference and overexpression of *jp* caused a reduction of lifespan from 53 days in control flies to only 35 days (Fig. 3A). Mean survival was also reduced from 47 days in control flies to 27 days in OE flies and only 17 days in KD flies. In this survival curve, it is evident that the lifespan of the control flies is very reduced compared with other survival curves in this work (Fig. 2A, Fig. 4A). This reduction is attributable to culture at 29°C, which is a suboptimal temperature but allows a better development of cardiac phenotypes.

To study heart function, cardiac contractions were analysed in 1-week-old adult fly hearts. There was no alteration of the diastolic interval (DI), the systolic interval (SI) or heart period length (HP, defined as DI+SI) (Fig. S3). The arrhythmia index (AI), an indicator of the variability calculated by dividing the standard deviation of the heart period by its median, was also unaltered (Fig. S3).

By contrast, we found changes in cardiac chamber parameters, including increased end-systolic diameter (ESD) and end-diastolic diameter (EDD) and decreased fractional shortening (FS) percentage, which provides an indication of the cardiac output (Fig. 3B-B″). These changes are evident in the M-mode traces of the three genotypes (Fig. 3C). These data revealed that in flies with altered levels of Jp expression, the heart tube is dilated and there is a dysfunction of the contractile properties that reduces cardiac output. For a more detailed observation of heart morphology in the *jp* mutants, we examined transverse semi-thin sections of the heart tube (Fig. 3D-D″). We observed an enlargement of the cardiac chamber, which we have previously quantified as an increased EDD (Fig. 3B′). Notably, these sections show that the thickness of the heart wall in the mutant flies does not show any statistically significant difference from control flies, discounting hypertrophy of cardiomyocytes (Fig. 3E).

*Drosophila* heart tubes have two types of muscle fibres, each with a distinct myofibrillar structure (Mery et al., 2008; Taghli-Lamallem et al., 2008); spirally or transversely oriented myofibrils that represent the contractile ‘working’ myocardium, and longitudinally oriented myofibrils that are found along the ventral surface of the tube (Molina and Cripps, 2001). In young flies, both types of myofibrils exhibit a tight and well-aligned arrangement. Cardiac myofibrils stain uniformly along the entire length of the thin filament with phalloidin (Ao and Lehrer, 1995), which can therefore be used to visualize both types of myofibrils. Phalloidin staining of actin in mutant flies with altered expression of *jp* revealed structural abnormalities in the parallel alignment of transverse myofibrils in the heart tube in the areas surrounding the ostia (Fig. 3D-D″). The cardiac fibres in the mutant flies were clearly more disorganized and less compact than in control flies. The gaps in myofibrillar staining were quantified by measuring the size of these areas in confocal stacks of five hearts of each genotype (Fig. 3G). The percentage area devoid of myofibrils was significantly smaller in control hearts compared with that of KD or OE (2, 10 and 7.8%, respectively).

### Modification of the Jp levels produces neurological abnormalities and affects the number of photoreceptor neurons

To evaluate the neuronal relevance of Jp in *Drosophila*, we used the pan-neuronal *elav-Gal4* driver. The control genotype was *elav-Gal4/+*, the OE genotype *elav-Gal4/UAS-jp*, and the KD genotype *elav-Gal4/UAS-jp^RNAi^*. Mean survival was strongly reduced in OE flies, whereas a slight reduction was observed in the KD flies (Fig. 4A). The bang-sensitive phenotype, a temporary paralysis when exposed to mechanical stress, has been associated with mutations in genes involved in neuronal function (Graham et al., 2010; Kuebler and Tanouye, 2000; Lee and Wu, 2002; Pavlidis et al., 1994; Schubiger et al., 1994; Trotta et al., 2004). Bang-sensitivity analyses revealed that KD flies exhibit a two- to threefold increase in recovery time at 1 week and at least a fivefold increase at 4 weeks compared with control flies (Fig. 4B). The recovery time of the OE flies was also increased, and the difference was statistically significant at 4 weeks.

The fly retina is a tissue widely used to study neurodegeneration. To drive expression in the retina, we used the *GMR-Gal4 (I)* construct (control genotype *GMR-Gal4/+*, OE genotype *GMR-Gal4/+; UAS-jp/+*, and the KD genotype *GMR-Gal4/+; UAS-jp^RNAi^/+*). In the control eyes, we can observe the wild-type external morphology; the ommatidial lenses are dome shaped and arranged in a hexagonal tiling pattern with inter-ommatidial bristles (Fig. 4C,C′). Overexpression of Jp produces a mildly disrupted arrangement, with the presence of nonhexagonal ommatidia and supernumerary bristles (Fig. 4D,D′). By contrast, KD resulted in no observable abnormalities in the external morphology (Fig. 4E,E′). Although *GMR-Gal4; UAS-jp^RNAi^* on its own has no phenotype, it is able to correct the phenotype of the eye external morphology caused by overexpression of *UAS-jp* under the control of *GMR-Gal4*, which demonstrates the specificity of the abnormal phenotype attributable to the overexpression of *jp* (Fig. 4F,F′).

A cross-section of a normal ommatidium always cuts through seven rhabdomeres, the light-collecting organs of the neuron, in a stereotypical trapezoidal arrangement (Fig. 4G). Transmission electron microscopy analyses of OE and KD retinas revealed an abnormal number of photoreceptors in both genotypes (Fig. 4G-J). Almost 50% of the OE ommatidia had extra photoreceptor cells, whereas in KD flies the effect was the opposite, with several ommatidia containing fewer photoreceptor cells (Fig. 4I). The presence of extra photoreceptor cells has been extensively described as a consequence of signalling defects during the development in the eye, whereas loss of photoreceptor cells could be a result of defects in such signalling pathways but also neurodegenerative processes, even at early post-eclosion stages, or a mixture of them. To discriminate between these situations, we performed the following experiment. To reduce the expression of the *jp RNAi* during development, flies were crossed and reared at 18°C until eclosion. At this point, the flies were divided into two groups; one was kept for 1 day at 18°C the other cultured for 7 days at 25°C after eclosion to allow stronger expression of the RNAi. In KD flies that were kept at 18°C, the morphology is slightly compromised, probably as a result of incipient degeneration, because RNAi expression is damped, not abolished, but at this point most ommatidia have seven photoreceptors. KD flies that were moved to 25°C have an enhanced loss of photoreceptors and a more degenerative morphology, with many vacuoles (Fig. S4). Therefore, whereas overexpression of *jp* results in recruitment of extra photoreceptor neurons, *jp* RNAi results mainly in neurodegeneration rather than photoreceptor specification.

### The Htt-related neurodegeneration is modified by altering the Jp levels

Dominant mutations in the *JPH3* gene caused by an expanded CAG/CTG repeat in its alternatively spliced exon 2A are responsible for Huntington's disease-like 2 (HDL2), a phenocopy clinically indistinguishable from Huntington's disease (HD) (Holmes et al., 2001). HD is a fatal neurodegenerative condition caused by expansion of the polyglutamine tract in the Huntingtin (Htt) protein, and the precise disease manifestations and their timing are affected by modifier genes (Gusella and MacDonald, 2009). Despite the phenotypical similarities, a possible role of JPHs as genetic modifiers in HD has not been investigated.

**Fig. 5.**
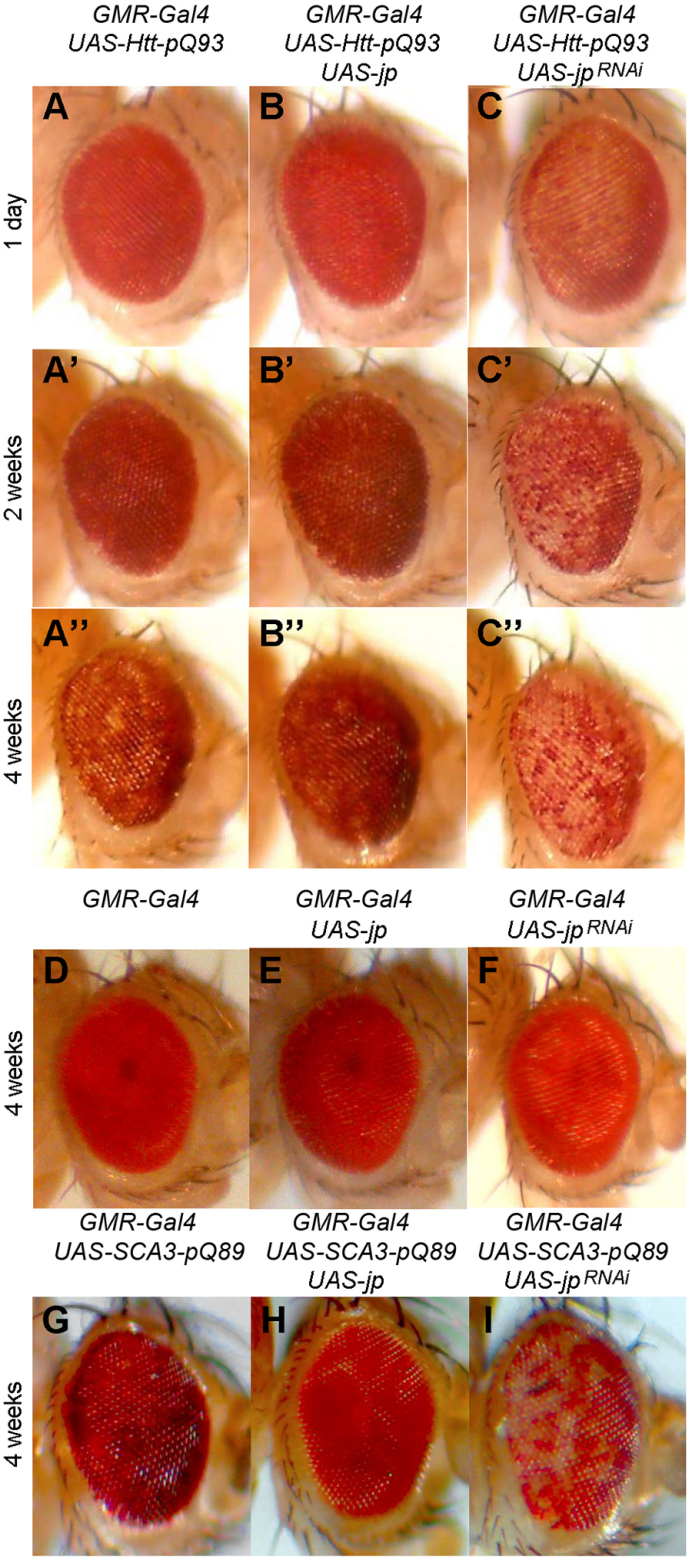
**Jp levels modify the phenotype of pathological Htt and SCA3 poly-Q expansions.** (A-A″) Progressive degeneration and depigmentation over 4 weeks upon expression of Htt-pQ93 in the eye driven by *GMR-Gal4*. (B-B″) Coexpression of Jp ameliorates the depigmentation. (C-C″) Coexpression of *jp* RNAi induces earlier, faster progressing depigmentation. *GMR-Gal4* alone (D), *GMR-Gal4*-driven *UAS-jp* (E) or *UAS-jp^RNAi^* (F) does not produce any depigmentation at 4 weeks. (G) Expression of SCA-Q89 produces eye depigmentation that is evident at 4 weeks. (H) Coexpression of Jp reduces depigmentation. (I) Coexpression of *jp* RNAi increases depigmentation.

HD has been modelled several times in *Drosophila* by overexpression of pathological expansions under neuronal drivers (Lewis and Smith, 2016), and these models have similar phenotypes to ours, having reduced lifespan when expressed under *elav-Gal4* and retinal neuron degeneration under *GMR-Gal4* (Fig. 4); and in addition, they also show features typical of HD, such as protein aggregates. The abnormal phenotypes in these models are repeat-length dependent. To model HD in *Drosophila*, we used a construct for the expression of human *HTT* exon 1 containing expanded polyglutamine repeats (*Htt-Ex1-pQ93*) that has been demonstrated to induce neurodegeneration (Steffan et al., 2001). These authors describe that shorter repeats (1Q or 20Q) have no deleterious effect. In order to investigate whether Jp could modify the HD pathogenesis in flies, *UAS-jp* or *UAS-jp^RNAi^* was coexpressed with *Htt-Ex1-pQ93*. Flies expressing *Htt-Ex1-pQ93* have a normal external morphology of the eye at day 1 post-eclosion, but suffer progressive degeneration of the underlying retina, resulting in patchy depigmentation after 4 weeks of age (Fig. 5A-A″). When *jp* is coexpressed with *Htt-Ex1-pQ93*, the development of this depigmentation is delayed (Fig. 5B-B″), which means that Jp might act as a partial suppressor of the *Htt-Ex1-pQ93*-induced degeneration. Conversely, coexpression of *Htt-Ex1-pQ93* with the *jp RNAi* led to enhancement of eye phenotype, because the loss of pigmentation is observed from day 1, and the depigmentation progresses much faster (Fig. 5C-C″). As a control experiment, we aged flies of the *GMR-Gal4*/*+*, *GMR-Gal4*; *UAS-jp* and *GMR-Gal4*; *UAS-jp^RNAi^* and observed no changes in pigmentation; therefore, the results observed were bona fide genetic modifications.

The *Htt-Ex1-pQ93* corresponds to a truncated version of the gene coding for only a few endogenous amino acids, so it is possible that *jp* functions as a modifier for other types of pathogenic poly-Q expansions. To test this possibility, we investigated whether *UAS-jp* or *UAS-jp^RNAi^* was able to modify the phenotype caused by expression of another dominant mutation caused by a poly-Q expansion in the human *SCA3* gene causative of spinocerebelar ataxia type 3 (Stochmanski et al., 2012). *UAS-SCA3-Q89* contains an expanded tract of glutamines within a full-length *SCA3* cDNA, and it also causes depigmentation of the *Drosophila* retina when expressed under *GMR-Gal4* (Fig. 5G). Again, coexpression of *UAS-jp* slows down depigmentation at 4 weeks (Fig. 5H), whereas coexpression of *UAS-jp^RNAi^* enhances it (Fig. 5I). The modification of the phenotype is clear, suggesting that mechanistically Jp can be a modifier of poly-Q expansions in general, but this does not necessarily mean that the modification is clinically relevant, because SCA3 and HD/HDL2 affect different regions of the encephalon.

### Jp has functional interactions with the Notch pathway

**Fig. 6.**
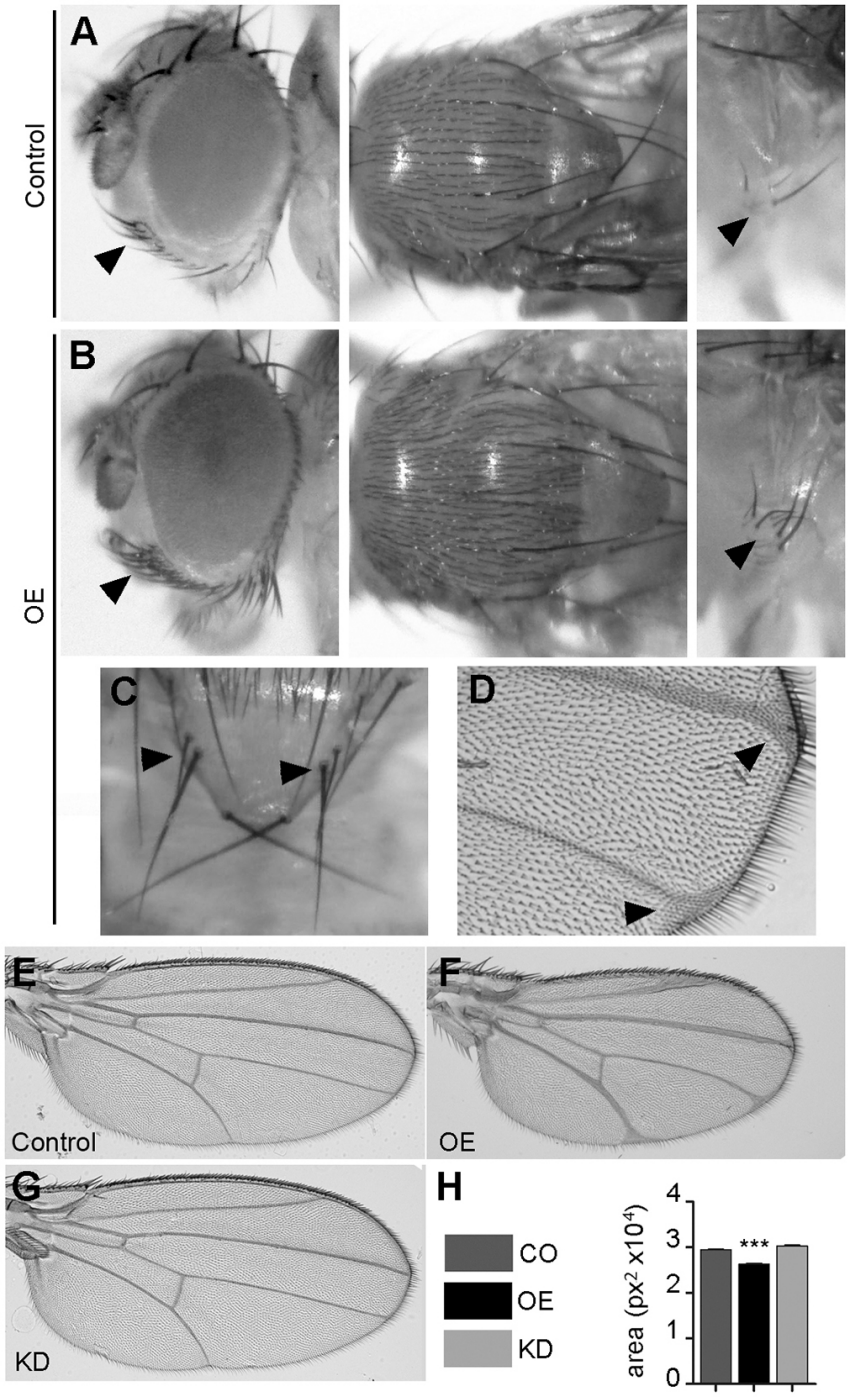
**Notch-like phenotypes in OE flies.** (A) Detail of the normal number and distribution of head vibrissae, notum microchaetae and sternopleural bristles in a control *Act-Gal4*/*+* fly. (B) Bristles of these types are increased in number in flies of the *Ac-Gal4*, *UAS-jp* genotype (OE). (C) Duplicated scutellar macrochaetae in an OE fly. (D) Delta-shaped contacts of the wing veins with the wing margin in an OE fly. (E) Morphology of a wild-type control fly wing (*rn-Gal4*/+). (F) *rn-Gal4*, *UAS-jp* fly wing with expanded wing veins, delta-shaped contacts with the wing margin and decreased wing blade size. (G) *rn-G*al4, *UAS-jp^RNAi^* wings have wild-type morphology. (H) Quantification of the blade area in wings of the genotypes shown in E-G shows that only OE wings are significantly different from the control (*n*≥11). In bar diagrams, data are represented as mean±s.e.m. One way-ANOVA, ***P<0.001.

A closer examination of the normally viable *Act5C-Gal4/UAS-jp* individuals revealed abnormal phenotypes usually linked to a downregulation of the Notch signalling pathway, such as supernumerary vibrissae under the eye, microchaetae in the notum and sternopleural bristles (Fig. 6A,B) (Schweisguth and Posakony, 1994). Further Notch phenocopies are the duplication of the macrochaetae in the scutellum (Fig. 6C) and delta-shaped wing veins (Fig. 6D). All these phenotypes were 100% penetrant. The phenotype we observed under OE of *jp* in the retina also points to a downregulation of Notch signalling, because the recruitment of extra cells as photoreceptor neurons is typical of mutants for the Notch ligand Delta (Parks et al., 1995). If this was a result of a functional interaction between Jp and the Notch pathway, either direct or indirect, the prediction would be that *jp* OE should enhance Notch phenotypes and *jp* KD should suppress them. To carry out these tests, we could not use the *Act5C-Gal4* driver with which we detected the phenotypes, because *jp* KD with this driver is lethal before the adult age. We also discarded the retina as an experimental tissue, because Notch signalling has successive and complex roles during eye development, including growth, planar cell polarity and several rounds of cell-type specification (Cagan and Ready, 1989). The wing blade is a more suitable model to investigate modification of the Notch/Delta phenotypes; therefore, we used two *Gal4* lines that drive expression in the whole wing blade, *rn-Gal4 (III)* and *nub-Gal4 (II)*.

**Fig. 7.**
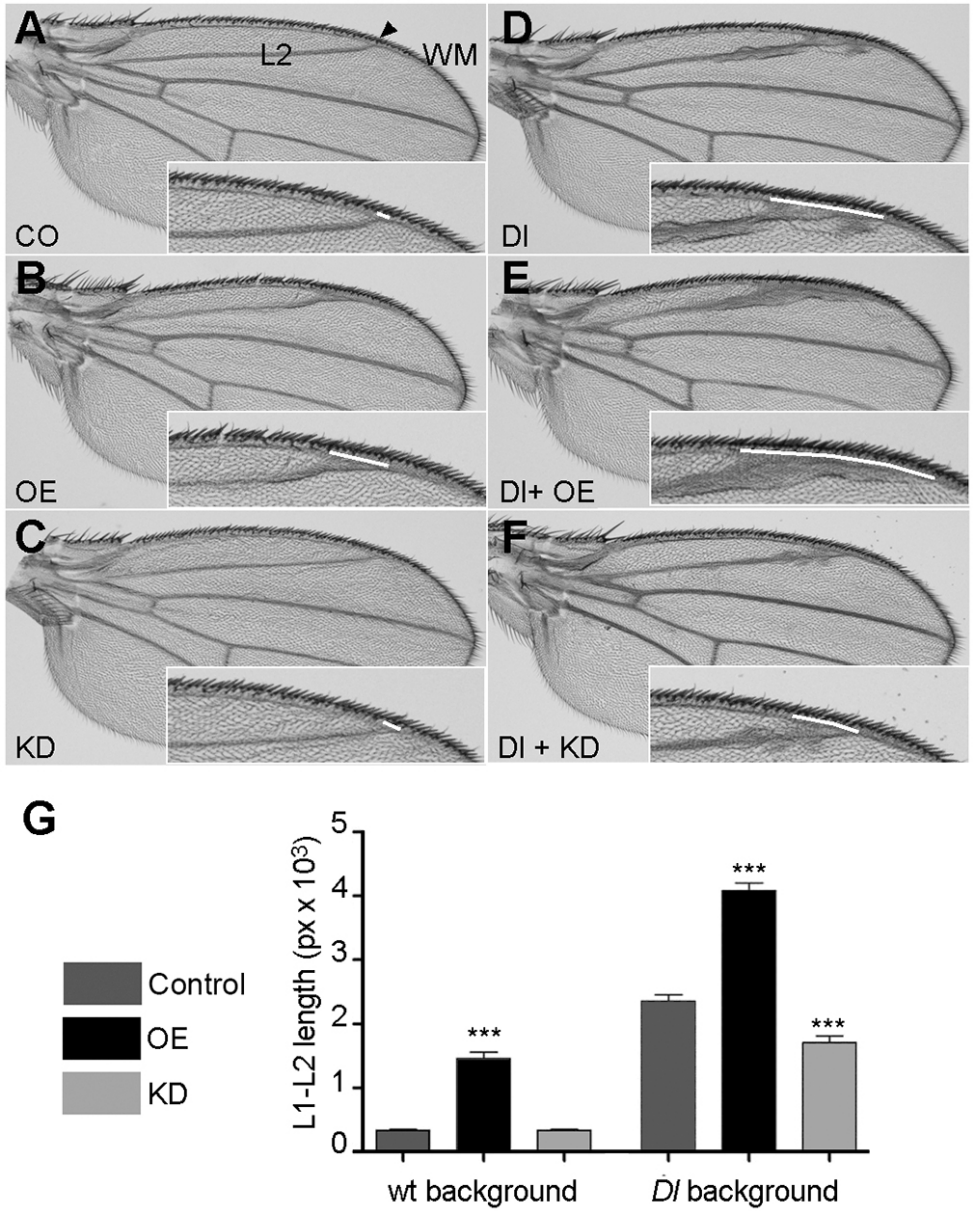
**Levels of Jp expression modify the phenotype of a *Dl* mutant allele.** (A-C) At 29°C, wing blades of control *nub-Gal4*/*+* (A), OE (B) and KD (C) genotypes have the same phenotypes as flies of the same genotypes cultured at the standard temperature. (D) Wing of a *Dl^6B37^* fly displaying the typical Dl phenotype of engrossed veins and delta-shaped contacts with the wing margin. (E) Overexpression of Jp in a *Dl^−^* background enhances the phenotype. (F) Expression of the *jp* RNAi in a *Dl^−^* background strongly suppresses the wing vein phenotypes. In each panel, the inset shows a higher magnification of the area where measurements were performed, and the white line indicates the length of the contact of vein L2 with the wing margin used in G. (G) Quantification of the length of the contact of vein L2 with the wing margin in flies of the genotypes represented in A-F (*n*≥17 for each genotype). In bar diagrams, data are represented as mean±s.e.m. One-way ANOVA, ***P<0.001.

Initially, we tested the effect of *jp* OE and KD on their own. OE of *jp* driven by *rn-Gal4* driver again mimics a Notch phenotype, with thickening of the longitudinal wing veins and delta-shaped contacts of the veins and the wing margin (De Celis, 2003), and also produces a reduction of the wing area (Fig. 6E,F,H). By contrast, *jp* KD did not produce any evident phenotypes in the wing morphology (Fig. 6E,G,H). Similar experiments with the other wing driver, *nub-Gal4*, produced equivalent results and also showed that *jp* KD can correct *jp* OE despite not having an effect on its own (Fig. S5). To test whether the extra vein tissue was produced by a reduction in the activation of the Notch pathway, we attempted the modification of the phenotype of a dominant temperature-sensitive allele of the gene encoding the Notch ligand Delta, *Dl^6B37^*. These experiments were performed at 29°C, the temperature at which this *Dl* allele shows a more pronounced phenotype than in the normal rearing conditions at 25°C. In these conditions, again OE wings have a *Dl*-like phenotype and KD wings are normal (Fig. 7A,B,C). *Dl^6B37^* flies displayed the expected wing vein defects, which are most evident in the L2 vein, and delta-shaped contacts with the wing margin (Fig. 7D). Expression of Jp in the wing blade enhanced these phenotypes (Fig. 7E), whereas expression of the *jp* RNAi corrected them (Fig. 7F). In order to have a parameter that allowed for a statistical treatment, we measured the length of the contact of L2 with the wing margin, which is wider when it adopts the delta shape. These analyses confirmed that *jp* behaves as it would be expected from a typical modifier: OE of *jp* enhances the *Dl* phenotype and KD partly suppresses it (Fig. 7G).

## DISCUSSION

### *Drosophila* as a model to study Junctophilin function

In the present work, we present data indicating that *Drosophila* is a good model to study pathologies resulting from mutations in human JPH genes. Our two experimental genotypes are based on a gain and a loss of function, OE and KD, respectively. In general, the specific phenotypes we observe in a particular tissue are also opposite (i.e. the effect on Htt expansions or Notch wing phenotypes), although some of the nonspecific phenotypes, such as overall viability or motility, can be altered in the same sense in both. This reduced fitness indicates that a proper balance in the expression levels of *jp* is required for normal calcium homeostasis cell functions. The phenotypes we have described in muscle, heart and neurons are compatible with what has been described in patients with pathological mutations in JPH genes or in KO mouse models of these genes. In addition, we describe two new functional relationships that might be relevant in the pathogenesis of junctophilins with pathological poly-Q expansions in Huntington's disease and with Notch signalling.

### Junctophilin function in the heart and muscle

Down- and upregulation of *jp* expression mainly affects the structure of the diads in the IFMs. Elevated levels induce the formation of elongated SR cisternae and, less often, incorrect localization of the diad next to the myofibrils. This effect is similar to the one observed upon elevated expression of *Jhp1* in mouse heart (Komazaki et al., 2003). In these mice, cardiac diads had extended SR–T-tubule contacts. Overexpression of Jph2 in heart also increases the SR–T-tubule contacts (Guo et al., 2014). KD of *Drosophila jp* also produced phenotypes comparable to those observed in *Jph1* KO mice: incorrect formation of diads and vacuolated SR and T-tubules. The phenotypes in KD flies are dramatic, with rudimentary or nearly absent diads at 1 week and a fragmentation of the mitochondrial network at 4 weeks. This fragmentation in older flies could be related to the incipient mitochondrial damage observed in younger ones. In murine muscle, there is coexpression of Jph2, so our KD phenotype probably represents a more severe loss of function, thus unveiling that junctophilins are required for the proper formation of the SR–T-tubule contacts, not only their maturation.

In any case, neither our experimental genotypes nor the published KO or overexpression murine models show any structural degeneration of the myofibrils. This is confirmed in the metabolomic profile, because there were no alterations linked to muscular degeneration, only an imbalance in the carbohydrate levels, with increased glycogen and decreased trehalose. This result is confirmed by an independent work, in which a screening for genetic determinants of metabolic traits in whole flies revealed a genetic linkage of *jp* to energy metabolism traits and increased glycogen levels (Jumbo-Lucioni et al., 2010). A possible explanation for this deregulation of carbohydrate metabolism is an alteration of the insulin signalling pathway.

In fact, downregulation of the insulin pathway could also explain two unexpected phenotypes in the muscle KD flies: the extended lifespan and the muscle hypertrophy. Mitohormesis, the adaptive response of mitochondria to mild stress, has already been reported to produce an extended lifespan in *Drosophila* (Owusu-Ansah et al., 2013)*.* In this case, mitohormesis triggered by mild muscular stress and mitochondrial fragmentation resulted in lifespan extension, and this effect was mediated by the insulin pathway and the mitochondrial unfolded protein response. There are further examples of extended longevity caused by impairment of insulin signalling and/or mitochondrial stress (Wang and Hekimi, 2015; Zarse et al., 2012). As for muscle hypertrophy, it is usually assumed that low levels of branched-chain amino-acids (BCAA) are indicative of muscle deficiency, but there is a great heterogeneity and many factors impinging at different levels (Tom and Nair, 2006). One of the pathways involved is insulin signalling (Glass, 2005), and in this context, we observe significant alterations of glycogen and trehalose levels. This result could be because of the involvement of different pathways and deserves a deeper study.

Muscle hypetrophy was also observed in mouse skeletal muscle expressing Jph2 with the dominant negative mutations S165F and Y141H, associated with hypertrophic cardiomyopathy in patients (Woo et al., 2012, 2010). Several mutations in human JPH2 have been associated with hypertrophic cardiomyopathy (Beavers et al., 2014), and can cause a hypertrophyc phenotype when downregulated in mouse cardiac muscle cell lines (Landstrom et al., 2011). By contrast, the phenotype of the heart KD flies is more similar to a dilated cardiomyopathy, with increased ESD and EDD and reduced cardiac output. Recently, a new mutation in *JPH2* has been found to be associated with dilated cardiomhyopathy (Sabater-Molina et al., 2016). Therefore, mutations in *JPH2* could cause hypertrophic or dilated cardiomiopathy depending on factors such as the genetic background, degree of functionality of the mutant protein, or location of the mutation in a particular domain.

A difference between KD in muscle and heart is that in the latter, we could find structural alterations in the cardiac myofibrils. A possible explanation is that the muscular driver we used, *Mhc-Gal4*, is expressed in differentiated muscle, whereas the cardiac driver, *GMH5-Gal4*, contains a promoter region from the *tinman* gene, which drives expression throughout heart development from the early embryo. Alternatively, this difference could be attributable to intrinsic physiological differences between skeletal and cardiac muscle.

### Junctophilin function in neurons

A *Jph3* KO produces motor deficits in mouse models, and the double KO *Jph3/4* has a more severe phenotype, including the impairment of motor, learning and memory abilities (Moriguchi et al., 2006; Nishi et al., 2002; Seixas et al., 2012). Likewise, KD of Jp in *Drosophila* neurons also affects neuronal function as reflected in the bang-sensitivity test. Alteration of Jp in the retina revealed a mixture of neurodevelopmental defects and degeneration of the retinal neurons. Although neurodevelopmental defects probably involve the mis-regulation of the Notch signalling pathway during cell fate determination and differentiation stages, the neurodegeneration happens in fully differentiated neurons and is dependent on neural function or survival rather than development.

The modification of the *Htt-Ex1-pQ93* phenotype by Jp is clear cut; OE of Jp is a suppressor and KD an enhancer of the neurodegeneration. This modification is most probably functionally relevant, because ablation of *Drosophila* Htt exacerbates the neural toxicity elicited by the same construct we have used in the present work (Zhang et al., 2009). Also, neuronal store-operated calcium entry is a new therapeutic target for HD (Wu et al., 2011). Given that triplet expansions of JPH3 are causative of HDL2 (Holmes et al., 2001), the fact that altering Jp levels modifies the phenotype of flies expressing human *Htt-Ex1-pQ93* suggests that both proteins participate in at least one common cellular pathway. In addition to causing HDL2, it is possible that genetic variation in JPH3/4 participates in the clinical variability of HD patients.

A *Drosophila* model of HDL2 based on the expression of the human JPH3 mutant protein HDL2-Q138 showed that toxicity is attributable to the accumulation of the poly-Q-expanded protein in the nucleus, and this toxicity was alleviated by redirecting it to the cytoplasm (Krench et al., 2016). By contrast, HTT-Q138 aggregates remain cytoplasmic, which suggests that they do not share toxicity mechanisms. Although this finding might seem to contradict ours, it has been demonstrated that the pathogenicity of *JPH3* mutations is multifactorial and involves at least two effects, a toxic gain of function of the aggregates and a deficit in JPH3 function attributable to reduced expression levels (Seixas et al., 2012). Therefore, overexpression of the human HTT-Q138 protein and modulation of the endogenous Jp levels could be affecting different cellular mechanisms in the nucleus and the cytoplasm, respectively.

### Junctophilin antagonizes Notch signalling

Overexpression of Jp phenocopies the loss of function of the Notch ligand Dl: recruitment of supernumerary photoreceptor neurons, lateral inhibition in sensory organ determination and expansion of the wing veins. A role of Jp as a modifier of Notch is strongly reinforced by the fact that loss of function of Jp can suppress the mutant phenotype of a dominant loss of function allele of Dl. Although this was an unexpected result, there are three previous high-throughput screenings for modifiers of Notch phenotypes in the wing (Cruz et al., 2009; Molnar et al., 2006) or in the eye (Shalaby et al., 2009), in which *Drosophila jp* was among the identified candidates. All of them were based on overexpression of genes adjacent to insertions with bi-directional *UAS* promoters, either the same original insertion we have used in our work (*P{XP}jp^d04563^*) or a similar construct (*P-GS*). In consequence, the authors could not discern which of the two genes flanking the insertion, *jp* or *CG3838*, was the modifier. Given that we have reproduced the modification after removing the promoter pointing to *CG3838*, we can single out *jp* as the Notch-interacting gene.

Our work is the first report of a functional relationship of junctophilins with Notch signalling. The effect of Jp on Notch is most probably through its effect on calcium trafficking. The ER calcium sensor STIM1 has been shown to co-localize with JPH1 during store-operated calcium entry (Pla-Martin et al., 2015) and interacts physically with JPH4 (Woo et al., 2016). *Drosophila Stim* has also been demonstrated to be a modifier of Notch phenotypes (Eid et al., 2008), although in this case *Stim* expression is synergistic rather than antagonistic to the pathway. Another screening in *Drosophila* has unveiled other calcium signalling proteins, such as calmodulin or ryanodine receptor, as modifiers of presenilin-dependent Notch signalling (van de Hoef et al., 2009).

This intimate relationship of calcium and Notch signalling suggests that defects in Notch could contribute to the pathogenicity of Jp mutations. Disruption of *Drosophila* Notch pathway members results in a dilated cardiomyopathy similar to the one we describe in our models (Kim et al., 2010). As for the neural function of junctophilins, it is remarkable that the phenotypes of two mouse models, the *Jph3/4* double KO and a Notch antisense RNA, have a similar phenotype with impaired synaptic plasticity and long-term potentiation in hippocampal CA1 synapses (Moriguchi et al., 2006; Wang et al., 2004). In the light of all this evidence, the interplay of junctophilins and Notch mediated by calcium could prove to be a relevant disease mechanism in muscular and neural pathologies and deserves further attention.

## MATERIALS AND METHODS

### *Drosophila* stocks, maintenance and genetics

The following fly stocks were obtained from the Bloomington *Drosophila* Stock Center: *Oregon-R*, *w^1118^*, *Dl^6B37^*, *Act5C-Gal4*, *GMR-GAL4*, *Mhc-Gal4*, *Elav-Gal4*, *nub-GAL4*, *UAS-Ser*, *UAS-Dl*, *UAS-GFP*, *P{XP}Jp^d04563^* and *hs-FLP*; the RNAi line v100555 expressing a dsRNA for RNAi of *jp* (*CG4405*) and *UAS-Dcr2* were obtained from the Vienna *Drosophila* Resource Centre. Other drivers used were *GMH5-GAL4* (Wessells and Bodmer, 2004), *rn-GAL4* (St Pierre et al., 2002), *UAS-Htt-ex1-pQ93* (Steffan et al., 2001). *twi;Mef-GAL4* is a recombinant carrying both *twi-Gal4* and *Mef2-Gal4*. For the modification of *SCA3* expansions, we used *UAS-SCA3-Q89*, expressing a full-length cDNA (Stochmanski et al., 2012). Flies were maintained on standard cornmeal medium at 25°C unless stated otherwise in the Results section.

The *UAS-jp* line was obtained by removing one of the two UAS promoters pointing in opposite directions in the *P{XP}* transposon in the *P{XP}jp^d04563^* insertion, leaving only the UAS promoter pointing towards the *jp* gene. For these, we crossed to flies with that express the Flipase protein under the control of a heat shock promoter, *hs-FLP*. The removal of the UAS was confirmed by PCR and sequencing of the amplified fragment. The following oligonucleotides were used for the PCR: JP-CG4405-FLPout-F (TGCTGTGGTCCGTTCTCTTGGC) and JP-CG4405-FLPout-R (TCGGCTGCTGCTCTAAACGACG).

### Nucleic acid isolation and qPCR

Quick Fly Genomic DNA Prep protocol (Berkeley *Drosophila* Genome Project resources) was used to isolate gDNA for genotyping. The methods for the RNA isolation and the cDNA synthesis were previously described (López Del Amo et al., 2015). The qPCRs were performed with SYBR Green SuperMix (Quanta BioSciences, Beverly, MA, USA) in a LightCycler LC480 real-time PCR instrument (Roche, Basel, Switzerland). Each qPCR was performed in triplicate for all genotypes, and each individual sample was obtained by pooling 10 individual flies in the RNA extraction. The relative mRNA levels were calculated according to the 2^−ΔΔ*Ct*^ method. Results were normalized to the expression of the *Gapdh* or *Rpl49* housekeeping genes.

For mitochondrial DNA copy number, total DNA was isolated as previously described (Scialò et al., 2015) and analysed by qPCR using primers against *mt:CoxI* (for mtDNA) and *Rpl32* (nuclear DNA, single-copy, for normalization).

### Lifespan and behavioural assays

For lifespan experiments, flies were collected using CO_2_ anaesthesia within 24-48 h of eclosion and then kept at a density of 20-25 flies per vial at 25°C (29°C in the case of the *GMH5-GAL4* driver). Flies were transferred to new vials every 2-3 days, and the number of dead flies was recorded. Lifespan studies were performed with a minimum of 50 flies from three independent experiments.

To examine locomotor ability, the flies were knocked down to the bottom of the vial by quick, firm tapping, and the proportion of flies that had climbed over the 9 cm mark within 10 s was determined. This assay was performed in triplicate for each genotype; at least 15 flies were used per genotype. For the flight assay, individual flies were transferred to a Petri dish, then the lid was removed and the dish inverted over a 45-cm-long cylinder and gently tapped to loosen the fly. Flies were either able to stabilize their flight and stay at the wall of the vessel (this position was scored in centimetres) or fell at the base and were scored as 45 cm. Thirty flies were scored for each genotype. The bang­-sensitivity assay was performed as previously described (Graham et al., 2010). A minimum of 10 flies from four independent experiments were tested for any particular genotype.

### Cardiac physiological analysis

For the physiological analysis, female flies were collected immediately after eclosion and maintained for 7 days at 29°C. For the heart beat recordings, semi-intact heart preparations and semi-automated optical heartbeat analysis were carried out as previously described (Chakraborty et al., 2015; Ocorr et al., 2007). A minimum of 15 hearts were analysed per genotype.

### Histology and microscopy

Flies were examined under an Olympus SZ60 stereomicroscope (Olympus, Tokyo, Japan) equipped with a Scopetek MDC200 Digital Camera (Hangzhou Scopetek Opto-Electric Co., Hangzhou, China). Areas (retina, IFM and wings) and lengths (L1-L2 wing veins) were measured using the ImageJ software (version 1.47; National Institutes of Health, Bethesda, MA, USA). Adult cuticles and wings were mounted in Hoyer's medium and analysed with a Leica DM6000 microscope.

Scanning electron microscopy (SEM) analysis of adult eyes was performed as previously described (Calpena et al., 2015), following the critical point drying method. Images were taken with a Philips XL-30 ESEM scanning electron microscope.

For light and transmission electron microscopy, eyes, thoraxes and abdomens were dissected and fixed overnight with 2% paraformaldehyde and 2% glutaraldehyde in 0.1 M phosphate buffer as previously described (López Del Amo et al., 2015). All samples were post-fixed in OsO_4_ for 2 h and dehydrated through an ethanol series; thereafter, samples were embedded in Durcupan epoxy resin (Sigma-Aldrich, St Louis, MO; USA). For transmission electron microscopy, 80-nm-thick sections were stained with uranyl acetate and examined with an FEI Tecnai Spirit G2 microscope. For bright field microscopy, 1.5 µm sections of thoraxes and abdomens were stained with Toluidine Blue, and images were examined with a Leica DM6000 microscope. Thorax sections were used for assessment of muscle defects; for the muscle section area, a minimum of six flies per genotype were analysed. Abdomen sections were used to evaluate heart wall thickness. A minimum of 10 sections from different individuals were analysed, and three different measurements of wall thickness were performed on each section. The number of photoreceptor neurons per ommatidium was determined by analysing transmission electron microscopy images of sections of retina from 1-week-old flies. For each genotype, sections from three different individuals were studied, and at least 60 ommatidia were scored in each section.

For confocal microscopy, fly hearts were dissected from 7-day-old females, fixed for 20 min in 4% paraformaldehyde, washed in PBT (PBS containing 0.3% Triton X-100), and stained with phalloidin for 20 min as previously described (Chakraborty et al., 2015). All confocal images were obtained with an Olympus FV1000 microscope. The gaps in myofibrillar staining were quantified by measuring the size of these areas from confocal stacks of five hearts of each genotype using the ImageJ software (version 1.47). The percentage area devoid of myofibrils was calculated, and comparisons were made between the control hearts and the OE or KD genotypes.

### Mitochondrial circularity index

For the calculation of the circularity index of the mitochondria, the outline of the mitochondria was manually traced and then analysed with the ImageJ software (version 1.47). Sample size was three individuals per genotype; from each individual, two different muscle electron micrographs were analysed by scoring the index for 30 mitochondria per micrograph (a total of 180 mitochondria per genotype).

### NMR spectroscopy

For NMR spectroscopy, six samples were analysed for each one of the three genotypes, each one of them containing 15 flies. Sample preparation, NMR spectroscopy and data analysis were performed as described (López Del Amo et al., 2015).

### Statistical analysis

Data were analysed with Prism 5 (GraphPad). In the lifespan experiments, log-rank (Mantel–Cox) test was performed, and each one of the experimental genotypes was compared with the control. In the comparisons between the three genotypes at a single time point, we performed one-way ANOVA with Dunnett's multiple comparison test. Values shown represent means±s.e.m. In all figures, *P<0.05, **P<0.01 and ***P<0.001.

## Supplementary Material

Supplementary information

## References

[DMM029082C1] Ao X. and Lehrer S. S. (1995). Phalloidin unzips nebulin from thin filaments in skeletal myofibrils. *J. Cell Sci.* 108, 3397-3403. 8586652 10.1242/jcs.108.11.3397

[DMM029082C2] Beavers D. L., Wang W., Ather S., Voigt N., Garbino A., Dixit S. S., Landstrom A. P., Li N., Wang Q., Olivotto I. et al. (2013). Mutation E169K in junctophilin-2 causes atrial fibrillation due to impaired RyR2 stabilization. *J. Am. Coll. Cardiol.* 62, 2010-2019. 10.1016/j.jacc.2013.06.052 23973696 PMC3830688

[DMM029082C3] Beavers D. L., Landstrom A. P., Chiang D. Y. and Wehrens X. H. T. (2014). Emerging roles of junctophilin-2 in the heart and implications for cardiac diseases. *Cardiovasc. Res.* 103, 198-205. 10.1093/cvr/cvu151 24935431 PMC4809974

[DMM029082C4] Brand A. H. and Perrimon N. (1993). Targeted gene expression as a means of altering cell fates and generating dominant phenotypes. *Development* 118, 401-415. 8223268 10.1242/dev.118.2.401

[DMM029082C5] Cagan R. L. and Ready D. F. (1989). Notch is required for successive cell decisions in the developing Drosophila retina. *Genes Dev.* 3, 1099-1112. 10.1101/gad.3.8.1099 2792755

[DMM029082C6] Calpena E., Palau F., Espinós C. and Galindo M. I. (2015). Evolutionary history of the Smyd gene family in metazoans: a framework to identify the orthologs of human Smyd genes in Drosophila and other animal species. *PLoS ONE* 10, e0134106 10.1371/journal.pone.0134106 26230726 PMC4521844

[DMM029082C7] Chakraborty M., Selma-Soriano E., Magny E., Couso J. P., Perez-Alonso M., Charlet-Berguerand N., Artero R. and Llamusi B. (2015). Pentamidine rescues contractility and rhythmicity in a Drosophila model of myotonic dystrophy heart dysfunction. *Dis. Model. Mech.* 8, 1569-1578. 10.1242/dmm.021428 26515653 PMC4728315

[DMM029082C8] Chen B., Guo A., Zhang C., Chen R., Zhu Y., Hong J., Kutschke W., Zimmerman K., Weiss R. M., Zingman L. et al. (2013). Critical roles of junctophilin-2 in T-tubule and excitation-contraction coupling maturation during postnatal development. *Cardiovasc. Res.* 100, 54-62. 10.1093/cvr/cvt180 23860812 PMC3778961

[DMM029082C9] Chintapalli V. R., Wang J. and Dow J. A. T. (2007). Using FlyAtlas to identify better Drosophila melanogaster models of human disease. *Nat. Genet.* 39, 715-720. 10.1038/ng2049 17534367

[DMM029082C10] Cruz C., Glavic A., Casado M. and de Celis J. F. (2009). A gain-of-function screen identifying genes required for growth and pattern formation of the Drosophila melanogaster wing. *Genetics* 183, 1005-1026. 10.1534/genetics.109.107748 19737745 PMC2778956

[DMM029082C11] De Celis J. F. (2003). Pattern formation in the Drosophila wing: the development of the veins. *BioEssays* 25, 443-451. 10.1002/bies.10258 12717815

[DMM029082C12] Dietzl G., Chen D., Schnorrer F., Su K.-C., Barinova Y., Fellner M., Gasser B., Kinsey K., Oppel S., Scheiblauer S. et al. (2007). A genome-wide transgenic RNAi library for conditional gene inactivation in Drosophila. *Nature* 448, 151-156. 10.1038/nature05954 17625558

[DMM029082C13] Eid J.-P., Arias A. M., Robertson H., Hime G. R. and Dziadek M. (2008). The Drosophila STIM1 orthologue, dSTIM, has roles in cell fate specification and tissue patterning. *BMC Dev. Biol.* 8, 104 10.1186/1471-213X-8-104 18950512 PMC2584103

[DMM029082C14] Garbino A., van Oort R. J., Dixit S. S., Landstrom A. P., Ackerman M. J. and Wehrens X. H. T. (2009). Molecular evolution of the junctophilin gene family. *Physiol. Genomics* 37, 175-186. 10.1152/physiolgenomics.00017.2009 19318539 PMC2685503

[DMM029082C15] Glass D. J. (2005). Skeletal muscle hypertrophy and atrophy signaling pathways. *Int. J. Biochem. Cell Biol.* 37, 1974-1984. 10.1016/j.biocel.2005.04.018 16087388

[DMM029082C16] Graham B. H., Li Z., Alesii E. P., Versteken P., Lee C., Wang J. and Craigen W. J. (2010). Neurologic dysfunction and male infertility in Drosophila porin mutants: a new model for mitochondrial dysfunction and disease. *J. Biol. Chem.* 285, 11143-11153. 10.1074/jbc.M109.080317 20110367 PMC2856991

[DMM029082C17] Guo A., Zhang X., Iyer V. R., Chen B., Zhang C., Kutschke W. J., Weiss R. M., Franzini-Armstrong C. and Song L.-S. (2014). Overexpression of junctophilin-2 does not enhance baseline function but attenuates heart failure development after cardiac stress. *Proc. Natl. Acad. Sci. USA* 111, 12240-12245. 10.1073/pnas.1412729111 25092313 PMC4143026

[DMM029082C18] Gusella J. F. and MacDonald M. E. (2009). Huntington's disease: the case for genetic modifiers. *Genome Med.* 1, 80 10.1186/gm80 19725930 PMC2768966

[DMM029082C19] Hirata Y., Brotto M., Weisleder N., Chu Y., Lin P., Zhao X., Thornton A., Komazaki S., Takeshima H., Ma J. et al. (2006). Uncoupling store-operated Ca2+ entry and altered Ca2+ release from sarcoplasmic reticulum through silencing of junctophilin genes. *Biophys. J.* 90, 4418-4427. 10.1529/biophysj.105.076570 16565048 PMC1471867

[DMM029082C20] Holmes S. E., O'Hearn E., Rosenblatt A., Callahan C., Hwang H. S., Ingersoll-Ashworth R. G., Fleisher A., Stevanin G., Brice A., Potter N. T. et al. (2001). A repeat expansion in the gene encoding junctophilin-3 is associated with Huntington disease-like 2. *Nat. Genet.* 29, 377-378. 10.1038/ng760 11694876

[DMM029082C21] Ikeda A., Miyazaki T., Kakizawa S., Okuno Y., Tsuchiya S., Myomoto A., Saito S.-Y., Yamamoto T., Yamazaki T., Iino M. et al. (2007). Abnormal features in mutant cerebellar Purkinje cells lacking junctophilins. *Biochem. Biophys. Res. Commun.* 363, 835-839. 10.1016/j.bbrc.2007.09.062 17904530

[DMM029082C22] Ito K., Komazaki S., Sasamoto K., Yoshida M., Nishi M., Kitamura K. and Takeshima H. (2001). Deficiency of triad junction and contraction in mutant skeletal muscle lacking junctophilin type 1. *J. Cell Biol.* 154, 1059-1067. 10.1083/jcb.200105040 11535622 PMC2196186

[DMM029082C23] Jumbo-Lucioni P., Ayroles J. F., Chambers M. M., Jordan K. W., Leips J., Mackay T. F. C. and De Luca M. (2010). Systems genetics analysis of body weight and energy metabolism traits in Drosophila melanogaster. *BMC Genomics* 11, 297 10.1186/1471-2164-11-297 20459830 PMC2880307

[DMM029082C24] Kakizawa S., Moriguchi S., Ikeda A., Iino M. and Takeshima H. (2008). Functional crosstalk between cell-surface and intracellular channels mediated by junctophilins essential for neuronal functions. *Cerebellum* 7, 385-391. 10.1007/s12311-008-0040-1 18607668

[DMM029082C25] Kim I. M., Wolf M. J. and Rockman H. A. (2010). Gene deletion screen for cardiomyopathy in adult Drosophila identifies a new notch ligand. *Circ. Res.* 106, 1233-1243. 10.1161/CIRCRESAHA.109.213785 20203305 PMC2860286

[DMM029082C26] Kimball S. R. and Jefferson L. S. (2006). Signaling pathways and molecular mechanisms through which branched-chain amino acids mediate translational control of protein synthesis. *J. Nutr.* 136, 227S-231S. 16365087 10.1093/jn/136.1.227S

[DMM029082C27] Komazaki S., Ito K., Takeshima H. and Nakamura H. (2002). Deficiency of triad formation in developing skeletal muscle cells lacking junctophilin type 1. *FEBS Lett.* 524, 225-229. 10.1016/S0014-5793(02)03042-9 12135771

[DMM029082C28] Komazaki S., Nishi M. and Takeshima H. (2003). Abnormal junctional membrane structures in cardiac myocytes expressing ectopic junctophilin type 1. *FEBS Lett.* 542, 69-73. 10.1016/S0014-5793(03)00340-5 12729900

[DMM029082C29] Krench M., Cho R. W. and Littleton J. T. (2016). A Drosophila model of Huntington disease-like 2 exhibits nuclear toxicity and distinct pathogenic mechanisms from Huntington disease. *Hum. Mol. Genet.* 25, 3164-3177. 10.1093/hmg/ddw166 27288455 PMC5179919

[DMM029082C30] Kuebler D. and Tanouye M. A. (2000). Modifications of seizure susceptibility in Drosophila. *J. Neurophysiol.* 83, 998-1009. 10669511 10.1152/jn.2000.83.2.998

[DMM029082C31] Kumar S., Konikoff C., Van Emden B., Busick C., Davis K. T., Ji S., Wu L.-W., Ramos H., Brody T., Panchanathan S. et al. (2011). FlyExpress: visual mining of spatiotemporal patterns for genes and publications in Drosophila embryogenesis. *Bioinformatics* 27, 3319-3320. 10.1093/bioinformatics/btr567 21994220 PMC3223365

[DMM029082C32] Landstrom A. P., Weisleder N., Batalden K. B., Bos J. M., Tester D. J., Ommen S. R., Wehrens X. H. T., Claycomb W. C., Ko J.-K., Hwang M. et al. (2007). Mutations in JPH2-encoded junctophilin-2 associated with hypertrophic cardiomyopathy in humans. *J. Mol. Cell. Cardiol.* 42, 1026-1035. 10.1016/j.yjmcc.2007.04.006 17509612 PMC4318564

[DMM029082C33] Landstrom A. P., Kellen C. A., Dixit S. S., van Oort R. J., Garbino A., Weisleder N., Ma J., Wehrens X. H. T. and Ackerman M. J. (2011). Junctophilin-2 expression silencing causes cardiocyte hypertrophy and abnormal intracellular calcium-handling. *Circ. Heart Fail.* 4, 214-223. 10.1161/CIRCHEARTFAILURE.110.958694 21216834 PMC3059380

[DMM029082C34] Landstrom A. P., Beavers D. L. and Wehrens X. H. T. (2014). The junctophilin family of proteins: from bench to bedside. *Trends Mol. Med.* 20, 353-362. 10.1016/j.molmed.2014.02.004 24636942 PMC4041816

[DMM029082C35] Lee J. and Wu C. F. (2002). Electroconvulsive seizure behavior in Drosophila: analysis of the physiological repertoire underlying a stereotyped action pattern in bang-sensitive mutants. *J. Neurosci.* 22, 11065-11079. 12486202 10.1523/JNEUROSCI.22-24-11065.2002 PMC6758420

[DMM029082C36] Lewis E. A. and Smith G. A. (2016). Using Drosophila models of Huntington's disease as a translatable tool. *J. Neurosci. Methods* 265, 89-98. 10.1016/j.jneumeth.2015.07.026 26241927

[DMM029082C37] Li H., Ding X., Lopez J. R., Takeshima H., Ma J., Allen P. D. and Eltit J. M. (2010). Impaired Orai1-mediated resting Ca2+ entry reduces the cytosolic [Ca2+] and sarcoplasmic reticulum Ca2+ loading in quiescent junctophilin 1 knock-out myotubes. *J. Biol. Chem.* 285, 39171-39179. 10.1074/jbc.M110.149690 20937810 PMC2998103

[DMM029082C38] Li L., Pan Z.-F., Huang X., Wu B.-W., Li T., Kang M.-X., Ge R.-S., Hu X.-Y., Zhang Y.-H., Ge L.-J. et al. (2016). Junctophilin 3 expresses in pancreatic beta cells and is required for glucose-stimulated insulin secretion. *Cell Death Dis.* 7, e2275 10.1038/cddis.2016.179 27336719 PMC5143404

[DMM029082C39] López Del Amo V., Seco-Cervera M., García-Giménez J. L., Whitworth A. J., Pallardó F. V. and Galindo M. I. (2015). Mitochondrial defects and neuromuscular degeneration caused by altered expression of Drosophila Gdap1: implications for the Charcot-Marie-Tooth neuropathy. *Hum. Mol. Genet.* 24, 21-36. 10.1093/hmg/ddu416 25122658

[DMM029082C40] López Del Amo V., Palomino-Schätzlein M., Seco-Cervera M., García-Giménez J. L., Pallardó F. V., Pineda-Lucena A. and Galindo M. I. (2017). A Drosophila model of GDAP1 function reveals the involvement of insulin signalling in the mitochondria-dependent neuromuscular degeneration. *Biochim. Biophys. Acta* 1863, 801-809. 10.1016/j.bbadis.2017.01.003 28065847

[DMM029082C84] Ma Y., Creanga A., Lum L. and Beachy P. A. (2006). Prevalence of off-target effects in Drosophila RNA interference screens. *Nature* 443, 359-363. 10.1038/nature05179 16964239

[DMM029082C41] Mery A., Taghli-Lamallem O., Clark K. A., Beckerle M. C., Wu X., Ocorr K. and Bodmer R. (2008). The Drosophila muscle LIM protein, Mlp84B, is essential for cardiac function. *J. Exp. Biol.* 211, 15-23. 10.1242/jeb.012435 18083727

[DMM029082C42] Minamisawa S., Oshikawa J., Takeshima H., Hoshijima M., Wang Y., Chien K. R., Ishikawa Y. and Matsuoka R. (2004). Junctophilin type 2 is associated with caveolin-3 and is down-regulated in the hypertrophic and dilated cardiomyopathies. *Biochem. Biophys. Res. Commun.* 325, 852-856. 10.1016/j.bbrc.2004.10.107 15541368

[DMM029082C43] Molina M. R. and Cripps R. M. (2001). Ostia, the inflow tracts of the Drosophila heart, develop from a genetically distinct subset of cardial cells. *Mech. Dev.* 109, 51-59. 10.1016/S0925-4773(01)00509-3 11677052

[DMM029082C44] Molnar C., López-Varea A., Hernández R. and de Celis J. F. (2006). A gain-of-function screen identifying genes required for vein formation in the Drosophila melanogaster wing. *Genetics* 174, 1635-1659. 10.1534/genetics.106.061283 16980395 PMC1667087

[DMM029082C45] Moriguchi S., Nishi M., Komazaki S., Sakagami H., Miyazaki T., Masumiya H., Saito S.-Y., Watanabe M., Kondo H., Yawo H. et al. (2006). Functional uncoupling between Ca2+ release and afterhyperpolarization in mutant hippocampal neurons lacking junctophilins. *Proc. Natl. Acad. Sci. USA* 103, 10811-10816. 10.1073/pnas.0509863103 16809425 PMC1502313

[DMM029082C46] Murphy R. M., Dutka T. L., Horvath D., Bell J. R., Delbridge L. M. and Lamb G. D. (2013). Ca2+-dependent proteolysis of junctophilin-1 and junctophilin-2 in skeletal and cardiac muscle. *J. Physiol.* 591, 719-729. 10.1113/jphysiol.2012.243279 23148318 PMC3577539

[DMM029082C47] Nishi M., Mizushima A., Nakagawara K. and Takeshima H. (2000). Characterization of human junctophilin subtype genes. *Biochem. Biophys. Res. Commun.* 273, 920-927. 10.1006/bbrc.2000.3011 10891348

[DMM029082C48] Nishi M., Hashimoto K., Kuriyama K., Komazaki S., Kano M., Shibata S. and Takeshima H. (2002). Motor discoordination in mutant mice lacking junctophilin type 3. *Biochem. Biophys. Res. Commun.* 292, 318-324. 10.1006/bbrc.2002.6649 11906164

[DMM029082C49] Nishi M., Sakagami H., Komazaki S., Kondo H. and Takeshima H. (2003). Coexpression of junctophilin type 3 and type 4 in brain. *Brain Res. Mol. Brain Res.* 118, 102-110. 10.1016/S0169-328X(03)00341-3 14559359

[DMM029082C100] Ocorr K., Perrin L., Lim H. Y., Qian L., Wu X. and Bodmer R. (2007). Genetic control of heart function and aging in Drosophila. *Trends Cardiovasc. Med.* 17, 177-182. 10.1016/j.tcm.2007.04.001 17574126 PMC1950717

[DMM029082C50] Owusu-Ansah E., Song W. and Perrimon N. (2013). Muscle mitohormesis promotes longevity via systemic repression of insulin signaling. *Cell* 155, 699-712. 10.1016/j.cell.2013.09.021 24243023 PMC3856681

[DMM029082C51] Parks A. L., Turner F. R. and Muskavitch M. A. T. (1995). Relationships between complex Delta expression and the specification of retinal cell fates during Drosophila eye development. *Mech. Dev.* 50, 201-216. 10.1016/0925-4773(94)00336-L 7619731

[DMM029082C52] Pavlidis P., Ramaswami M. and Tanouye M. A. (1994). The Drosophila easily shocked gene: a mutation in a phospholipid synthetic pathway causes seizure, neuronal failure, and paralysis. *Cell* 79, 23-33. 10.1016/0092-8674(94)90397-2 7923374

[DMM029082C53] Pla-Martin D., Calpena E., Lupo V., Márquez C., Rivas E., Sivera R., Sevilla T., Palau F. and Espinós C. (2015). Junctophilin-1 is a modifier gene of GDAP1-related Charcot-Marie-Tooth disease. *Hum. Mol. Genet.* 24, 213-229. 10.1093/hmg/ddu440 25168384

[DMM029082C54] Razzaq A., Robinson I. M., McMahon H. T., Skepper J. N., Su Y., Zelhof A. C., Jackson A. P., Gay N. J. and O'Kane C. J. (2001). Amphiphysin is necessary for organization of the excitation-contraction coupling machinery of muscles, but not for synaptic vesicle endocytosis in Drosophila. *Genes Dev.* 15, 2967-2979. 10.1101/gad.207801 11711432 PMC312829

[DMM029082C55] Reynolds J. O., Chiang D. Y., Wang W., Beavers D. L., Dixit S. S., Skapura D. G., Landstrom A. P., Song L.-S., Ackerman M. J. and Wehrens X. H. T. (2013). Junctophilin-2 is necessary for T-tubule maturation during mouse heart development. *Cardiovasc. Res.* 100, 44-53. 10.1093/cvr/cvt133 23715556 PMC3778955

[DMM029082C56] Sabater-Molina M., Navarro M., Garcia-Molina Saez E., Garrido I., Pascual-Figal D., Gonzalez Carrillo J. and Gimeno Blanes J. R. (2016). Mutation in JPH2 cause dilated cardiomyopathy. *Clin. Genet.* 90, 468-469. 10.1111/cge.12825 27471098

[DMM029082C57] Sarou-Kanian V., Joudiou N., Louat F., Yon M., Szeremeta F., Même S., Massiot D., Decoville M., Fayon F. and Beloeil J.-C. (2015). Metabolite localization in living drosophila using high resolution magic angle spinning NMR. *Sci. Rep.* 5, 9872 10.1038/srep09872 25892587 PMC4402646

[DMM029082C58] Schubiger M., Feng Y., Fambrough D. M. and Palka J. (1994). A mutation of the Drosophila sodium pump alpha subunit gene results in bang-sensitive paralysis. *Neuron* 12, 373-381. 10.1016/0896-6273(94)90278-X 8110464

[DMM029082C59] Schweisguth F. and Posakony J. W. (1994). Antagonistic activities of Suppressor of Hairless and Hairless control alternative cell fates in the Drosophila adult epidermis. *Development* 120, 1433-1441. 8050354 10.1242/dev.120.6.1433

[DMM029082C60] Scialò F., Sriram A., Naudí A., Ayala V., Jové M., Pamplona R. and Sanz A. (2015). Target of rapamycin activation predicts lifespan in fruit flies. *Cell Cycle* 14, 2949-2958. 10.1080/15384101.2015.1071745 26259964 PMC4630862

[DMM029082C61] Seixas A. I., Holmes S. E., Takeshima H., Pavlovich A., Sachs N., Pruitt J. L., Silveira I., Ross C. A., Margolis R. L. and Rudnicki D. D. (2012). Loss of junctophilin-3 contributes to Huntington disease-like 2 pathogenesis. *Ann. Neurol.* 71, 245-257. 10.1002/ana.22598 22367996

[DMM029082C62] Shalaby N. A., Parks A. L., Morreale E. J., Osswalt M. C., Pfau K. M., Pierce E. L. and Muskavitch M. A. T. (2009). A screen for modifiers of notch signaling uncovers Amun, a protein with a critical role in sensory organ development. *Genetics* 182, 1061-1076. 10.1534/genetics.108.099986 19448274 PMC2728848

[DMM029082C63] St Pierre S. E., Galindo M. I., Couso J. P. and Thor S. (2002). Control of Drosophila imaginal disc development by rotund and roughened eye: differentially expressed transcripts of the same gene encoding functionally distinct zinc finger proteins. *Development* 129, 1273-1281. 11874922 10.1242/dev.129.5.1273

[DMM029082C64] Steffan J. S., Bodai L., Pallos J., Poelman M., McCampbell A., Apostol B. L., Kazantsev A., Schmidt E., Zhu Y.-Z., Greenwald M. et al. (2001). Histone deacetylase inhibitors arrest polyglutamine-dependent neurodegeneration in Drosophila. *Nature* 413, 739-743. 10.1038/35099568 11607033

[DMM029082C65] Stochmanski S. J., Therrien M., Laganière J., Rochefort D., Laurent S., Karemera L., Gaudet R., Vyboh K., Van Meyel D. J., Di Cristo G. et al. (2012). Expanded ATXN3 frameshifting events are toxic in Drosophila and mammalian neuron models. *Hum. Mol. Genet.* 21, 2211-2218. 10.1093/hmg/dds036 22337953

[DMM029082C66] Taghli-Lamallem O., Akasaka T., Hogg G., Nudel U., Yaffe D., Chamberlain J. S., Ocorr K. and Bodmer R. (2008). Dystrophin deficiency in Drosophila reduces lifespan and causes a dilated cardiomyopathy phenotype. *Aging Cell* 7, 237-249. 10.1111/j.1474-9726.2008.00367.x 18221418 PMC2840698

[DMM029082C67] Takeshima H., Komazaki S., Nishi M., Iino M. and Kangawa K. (2000). Junctophilins: a novel family of junctional membrane complex proteins. *Mol. Cell* 6, 11-22. 10.1016/S1097-2765(05)00005-5 10949023

[DMM029082C68] Takeshima H., Hoshijima M. and Song L.-S. (2015). Ca^2+^ microdomains organized by junctophilins. *Cell Calcium* 58, 349-356. 10.1016/j.ceca.2015.01.007 25659516 PMC5159448

[DMM029082C69] Thibault S. T., Singer M. A., Miyazaki W. Y., Milash B., Dompe N. A., Singh C. M., Buchholz R., Demsky M., Fawcett R., Francis-Lang H. L. et al. (2004). A complementary transposon tool kit for Drosophila melanogaster using P and piggyBac. *Nat. Genet.* 36, 283-287. 10.1038/ng1314 14981521

[DMM029082C70] Tom A. and Nair K. S. (2006). Assessment of branched-chain amino Acid status and potential for biomarkers. *J. Nutr.* 136, 324S-330S. 16365107 10.1093/jn/136.1.324S

[DMM029082C71] Trotta N., Rodesch C. K., Fergestad T. and Broadie K. (2004). Cellular bases of activity-dependent paralysis in Drosophila stress-sensitive mutants. *J. Neurobiol.* 60, 328-347. 10.1002/neu.20017 15281071

[DMM029082C72] van de Hoef D. L., Hughes J., Livne-Bar I., Garza D., Konsolaki M. and Boulianne G. L. (2009). Identifying genes that interact with Drosophila presenilin and amyloid precursor protein. *Genesis* 47, 246-260. 10.1002/dvg.20485 19241393

[DMM029082C73] Wang Y. and Hekimi S. (2015). Mitochondrial dysfunction and longevity in animals: Untangling the knot. *Science* 350, 1204-1207. 10.1126/science.aac4357 26785479

[DMM029082C74] Wang Y., Chan S. L., Miele L., Yao P. J., Mackes J., Ingram D. K., Mattson M. P. and Furukawa K. (2004). Involvement of Notch signaling in hippocampal synaptic plasticity. *Proc. Natl. Acad. Sci. USA* 101, 9458-9462. 10.1073/pnas.0308126101 15190179 PMC438998

[DMM029082C75] Wessells R. J. and Bodmer R. (2004). Screening assays for heart function mutants in Drosophila. *BioTechniques* 37, 58-60, 62, 64 passim. 15283201 10.2144/04371ST01

[DMM029082C76] Woo J. S., Hwang J.-H., Ko J.-K., Weisleder N., Kim D. H., Ma J. and Lee E. H. (2010). S165F mutation of junctophilin 2 affects Ca2+ signalling in skeletal muscle. *Biochem. J.* 427, 125-134. 10.1042/BJ20091225 20095964

[DMM029082C77] Woo J. S., Cho C.-H., Lee K. J., Kim D. H., Ma J. and Lee E. H. (2012). Hypertrophy in skeletal myotubes induced by junctophilin-2 mutant, Y141H, involves an increase in store-operated Ca2+ entry via Orai1. *J. Biol. Chem.* 287, 14336-14348. 10.1074/jbc.M111.304808 22389502 PMC3340289

[DMM029082C78] Woo J. S., Srikanth S., Nishi M., Ping P., Takeshima H. and Gwack Y. (2016). Junctophilin-4, a component of the endoplasmic reticulum-plasma membrane junctions, regulates Ca2+ dynamics in T cells. *Proc. Natl. Acad. Sci. USA* 113, 2762-2767. 10.1073/pnas.1524229113 26929330 PMC4790987

[DMM029082C79] Wu J., Shih H.-P., Vigont V., Hrdlicka L., Diggins L., Singh C., Mahoney M., Chesworth R., Shapiro G., Zimina O. et al. (2011). Neuronal store-operated calcium entry pathway as a novel therapeutic target for Huntington's disease treatment. *Chem. Biol.* 18, 777-793. 10.1016/j.chembiol.2011.04.012 21700213 PMC3124661

[DMM029082C80] Xu M., Zhou P., Xu S.-M., Liu Y., Feng X., Bai S.-H., Bai Y., Hao X.-M., Han Q., Zhang Y. et al. (2007). Intermolecular failure of L-type Ca2+ channel and ryanodine receptor signaling in hypertrophy. *PLoS Biol.* 5, e21 10.1371/journal.pbio.0050021 17214508 PMC1764437

[DMM029082C81] Zarse K., Schmeisser S., Groth M., Priebe S., Beuster G., Kuhlow D., Guthke R., Platzer M., Kahn C. R. and Ristow M. (2012). Impaired insulin/IGF1 signaling extends life span by promoting mitochondrial L-proline catabolism to induce a transient ROS signal. *Cell Metab.* 15, 451-465. 10.1016/j.cmet.2012.02.013 22482728 PMC4844853

[DMM029082C82] Zhang S., Feany M. B., Saraswati S., Littleton J. T. and Perrimon N. (2009). Inactivation of Drosophila Huntingtin affects long-term adult functioning and the pathogenesis of a Huntington's disease model. *Dis. Model. Mech.* 2, 247-266. 10.1242/dmm.000653 19380309 PMC2675792

[DMM029082C83] Zhang H.-B., Li R.-C., Xu M., Xu S.-M., Lai Y.-S., Wu H.-D., Xie X.-J., Gao W., Ye H., Zhang Y.-Y. et al. (2013). Ultrastructural uncoupling between T-tubules and sarcoplasmic reticulum in human heart failure. *Cardiovasc. Res.* 98, 269-276. 10.1093/cvr/cvt030 23405000

