# Supplementary material for: The Drosophila junctophilin gene is functionally equivalent to its four mammalian counterparts and is a modifier of a Huntingtin poly-Q expansion and the Notch pathway
Source: Dis Model Mech. 2018 Jan 1;11(1):dmm029082. doi: 10.1242/dmm.029082 (PMC5818072; doi:10.1242/dmm.029082)
Supplement: Supplementary information [file dmm-11-029082-s1.pdf]

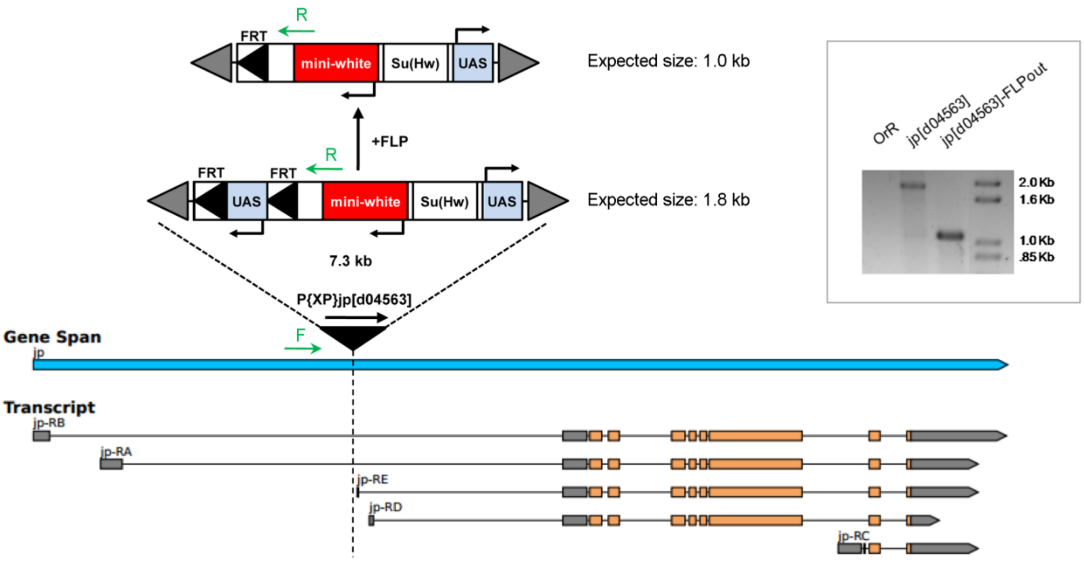

Figure S1. Strategy to generate the *UAS-jp* strain. Representation of the *P{XP}jp*^{d04563} insertion before and after FLP-mediated removal of the upstream *UAS* promoter. Forward (F) and reverse (R) priming sites are indicated by green arrows and the expected amplicon sizes are indicated next to each insertion. The gel shows the bands obtained in PCR amplification with genomic DNA of *OregonR* (negative control), and the two *P{XP}jp*^{d04563} versions.

Disease Models & Mechanisms • Supplementary information

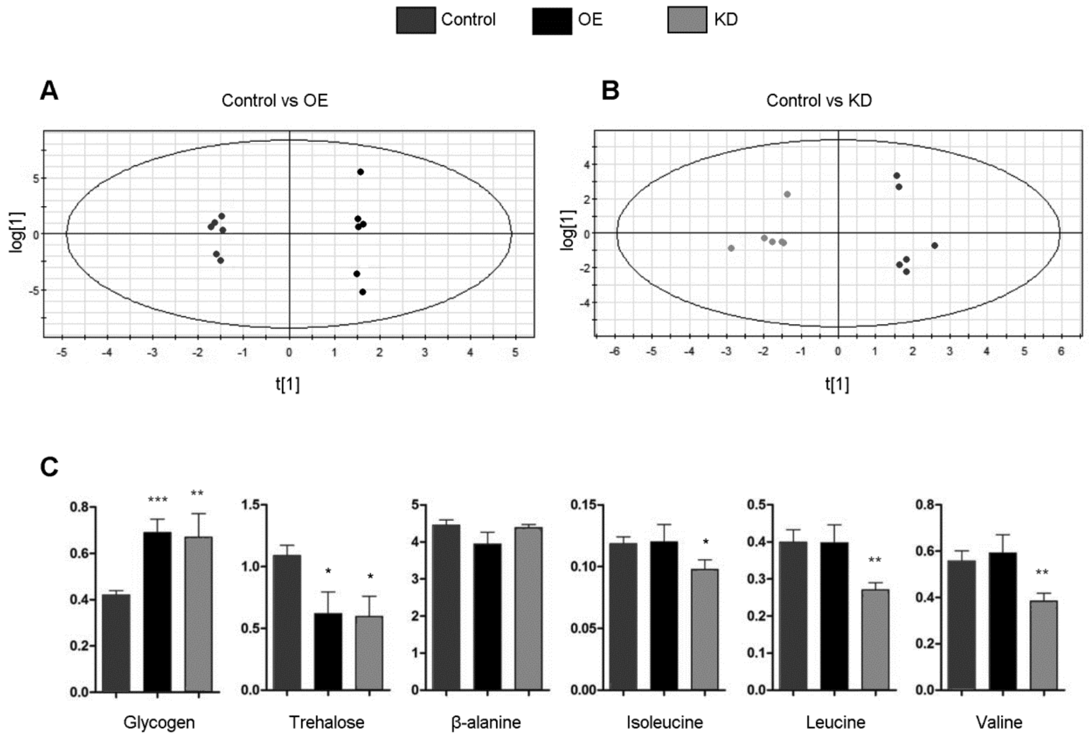

Figure S2. Metabolomic analysis of *Mhc-Gal4* OE and KD fly thoraxes. (A, B) OPLS-DA models allow discrimianion between the control and OE genotypes (A) and also between the control and KD genotypes (B). (C) relative abundance of metabolites, estimated as integrated peak area in the NMR spectrogram, showing statistically significant differences compared to the control

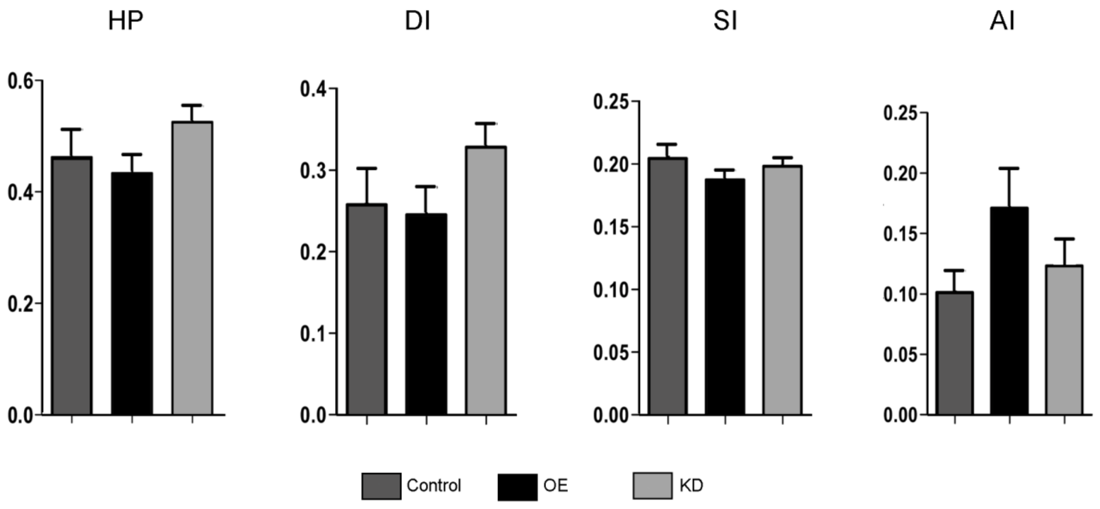

Figure S3. Unaffected cardiac parameters. Cardiac parameters that do not show any statistically significant differences with the control flies: heart period (HP), diastolic interval (DI), sistolic interval (SI) and arrhythmia index (AI), in parameters units are seconds.

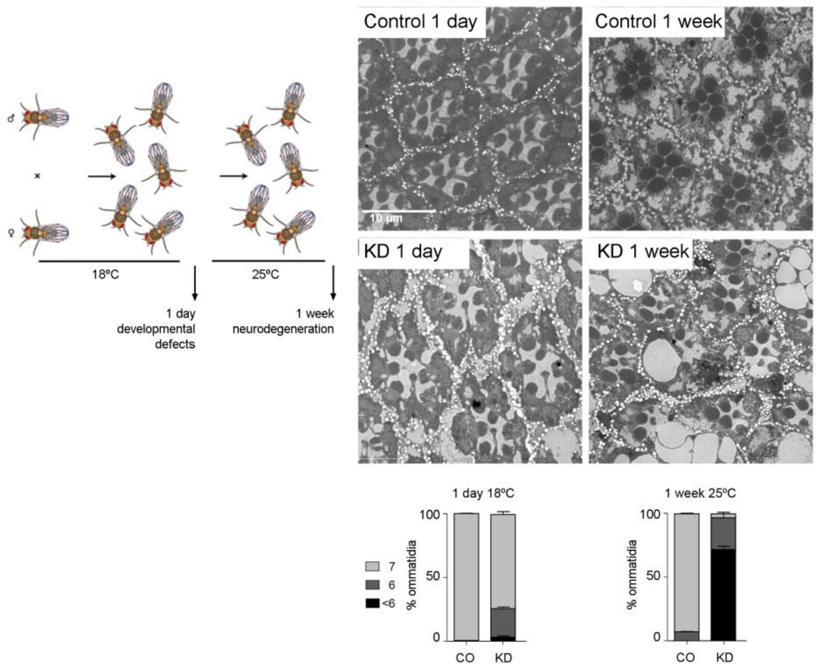

Figure S4. Analysis of neurodevelopmental vs neurodegenerative effects. To minimise developmental defects and highlight neurodegeneration, flies were cultured at 18ºC to tamper *GMR-Gal4* expression during development, and then for a week at 25ºC to get higher levels of Gal4 in differentiated retinal neurons. Retinas of control and KD flies were analysed at both time points, 1 day after eclosion and after 1 week at 25ºC. (n=3, ≥60 ommatidia each)

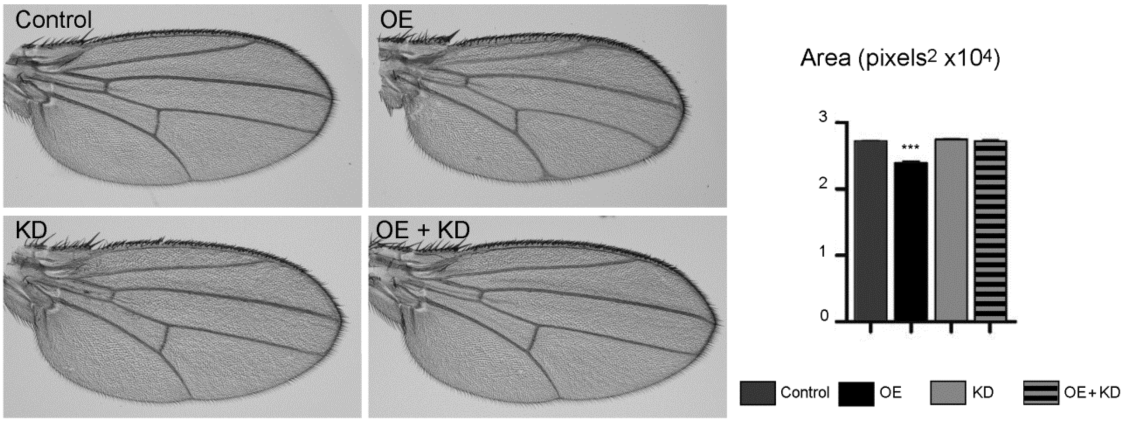

Figure S5. Alteration of Jp levels with *nub-Gal4*. The area of wing blades from flies bearing the *nub-Gal4* driver without any *UAS* construct (control), with *UAS-jp* (OE), with *UAS-jp*^{RNAi} (KD) or with both (OE + KD) was estimated. Only the OE wing blades have a statistical reduction in size, which is recovered by co-expression of *UAS*-jp^{RNAi} (n≥18; *** $p < 0.001$).
